# A bifunctional molecule-based strategy for the development of theranostic antibody-drug conjugate

**DOI:** 10.7150/thno.51232

**Published:** 2021-01-01

**Authors:** Dian Xiao, Lei Zhao, Fei Xie, Shiyong Fan, Lianqi Liu, Wei Li, Ruiyuan Cao, Song Li, Wu Zhong, Xinbo Zhou

**Affiliations:** National Engineering Research Center for the Emergency Drug, Beijing Institute of Pharmacology and Toxicology, Beijing 100850, China.

**Keywords:** theranostic, 7-amino-3-hydroxyethyl-coumarin, antibody-drug conjugate, self-elimination, on-off fluorescence

## Abstract

Antibody-drug conjugates (ADCs) are being developed worldwide with the potential to revolutionize current cancer treatment strategies. Developing novel theranostic ADCs with therapeutic utility and imaging capability is an attractive and challenging subject that promises advances in the field of personalized medicine. In this work, we propose a bifunctional molecule-based strategy for the development of theranostic ADCs.

**Methods:** We developed a theranostic ADC consisting of the anti-Her2 antibody Mil40, monomethyl auristatin E (MMAE) as the active payload, and a 7-amino-3-hydroxyethyl-coumarin (7-AHC)-based dipeptide linker, which functions as a novel bifunctional fluorescence probe that allows self-elimination cleavage in the presence of cathepsin B for payload release and fluorophore activation. The on-off fluorescence properties and the antitumor effect *in vitro* and *in vivo* were investigated.

**Results:** A 48-fold fluorescence enhancement was observed within 1 h when the 7-AHC-based linker was exposed to cathepsin B. Cleavage upon exposure to cathepsin B allows MMAE and fluorophore intracellular release and the monitoring of MMAE distribution using confocal microscopy. Additionally, the newly developed ADC retains the advantages of traditional p-aminobenzyloxycarbonyl-containing ADCs, such as good stability (t_1/2_ > 7 days) and high activity *in vitro* (IC_50_ = 0.09-3.74 nM). Importantly, the theranostic ADC exhibited the equivalent antitumor efficacy to the marketed ADC T-DM1 in the classic breast cancer model.

**Conclusion:** We suggest that the present strategy can be universally applied in all p-aminobenzyloxycarbonyl-containing ADCs. Overall, theranostic ADCs may play a role in developing new theranostic systems and promoting personalized medicine research.

## Introduction

Antibody-drug conjugates (ADCs) are exceptional target-specific prodrugs with the potential to revolutionize current treatment strategies and regimens for cancer 1, 2. An ADC is formulated by conjugating a toxic payload with a monoclonal antibody via a chemical linker. Presently, there are eight FDA-approved ADCs available to patients, with an additional 80 ADCs in different phases of clinical development 3, 4. The striking success has driven scientists and clinicians to improve this molecular platform for the development of effective therapeutics for microbial infection and immune modulation 5, 6.

Despite the great clinical success of ADCs, the key trafficking pathways involved in payload release remain unclear. Imaging studies have been performed with ADCs to address this issue. Although these studies have been very informative, they still have some drawbacks. The studies have mostly been conducted using fluorescence-labeled antibodies 7-9 or radiolabeled antibodies 10, which does not allow the follow-up of payload release. Moreover, the activity of the labeled ADC can be decreased more than 100 times. Recently, Lee *et al*. 11 developed a payload labeling method consisting in the addition of FRET reporter groups to both linker and payload. However, these modifications may lead to significant changes in the structure of the linker and payload, and the consequent impact on *in vivo* efficacy is not known. Michelle R. Sorkin *et al*. 12 developed a fluorescence-based antibody conjugate by directly replacing the payload with a fluorescent probe. This was tantamount to a totally new ADC. Overall, these studies may not truly reflect the intracellular drug release process, while the systems used may potentially lead to a loss in drug efficacy. Furthermore, a tedious process is required to prepare these labeled ADCs.

In recent years, theranostic systems have received increasing attention due to their potential therapeutic utility and imaging enhancement capability; however, these systems are still in an early phase of development 13-15. Our aim was to develop a novel theranostic ADC that could solve the abovementioned issues. Currently available fluorescent probes, such as the commonly used coumarins 16-18 (Figure S1), have high fluorescence quantum yield and good photostability but cannot be merged into the ADC structure due to their deficient self-elimination electron transfer property. Therefore, the perfect integration of fluorescence properties into ADCs has remained a challenge.

**Figure 1:**
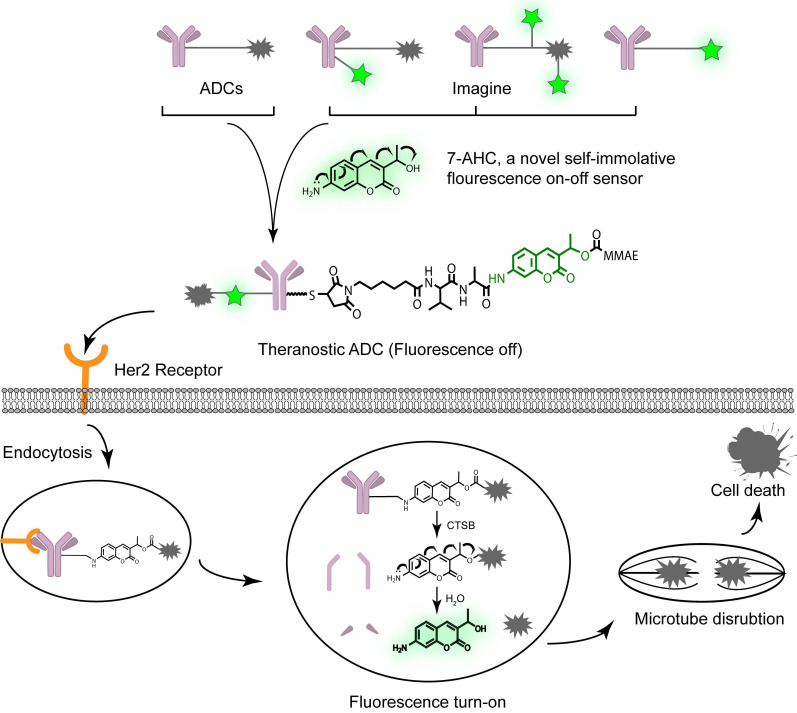
The design and proposed drug release mechanism of the theranostic ADC. The new self-elimination fragment 7-AHC was used to realize “all in one” design for targeting, imaging and therapy. Theranostic ADC targeted to the Her2 antigen and was internalized into tumor cells. And then the payload MMAE was released to kill tumor cells upon CTSB in lysosome. Simultaneously, the 7-AHC was activated to turn on the fluorescence.

In this study, we designed a novel bifunctional fluorescent probe, 7-amino-3-hydroxyethyl-coumarin (7-AHC), which demonstrated both self-elimination electron transfer and on-off fluorescence. Another advantage of 7-AHC is that it has similar properties (log P_PAB_ = 0.66, log P_7-AHC_ = 0.63) and functions to those of the standard self-elimination fragment p-aminobenzyloxycarbonyl (PAB) 19-21 group of successful ADCs (Figure S1). Given these characteristics, we further replaced PAB with this novel bifunctional molecule to develop a real theranostic ADC (Figure 1). The as-prepared structure looks like a traditional ADC but has “a fluorescent eye” that aids the scientific and visual understanding of the ADC release process, while retaining the advantages of traditional ADCs, such as high stability, long half-life, high activity, and other desired drug-like properties 21, 22.

To verify our proposal, we constructed a novel theranostic ADC using the anti-Her2 antibody Mil40, our new 7-AHC-based dipeptide (valine-alanine, VA) linker, and monomethyl auristatin E (MMAE), which is the most commonly used payload 23. In the presence of cathepsin B (CTSB), this novel ADC releases MMAE and the fluorophore 7-AHC. Thus, the enhanced fluorescence intensity after dipeptide bond cleavage can be observed, and 7-AHC serves as an excellent indicator of intracellular drug release. Simultaneously, the released MMAE exerts its toxicity depending on tubulin inhibition. To the best of our knowledge, this is the first report of a real theranostic ADC perfectly combining therapy and imaging. Furthermore, it can be used as a universal platform strategy for all PAB-containing linkers such as the valine citrulline (VCit) 24, 25 and VA 26, 27 linkers, along with various payloads such as MMAE 28, 29, SN38 30 and PBD 27. Beyond this study, theranostic ADCs may play a role in developing new theranostic systems and promoting personalized medicine research.

## Results and Discussion

### Synthesis of 7-AHC-VA-MMAE

**Figure 2:**
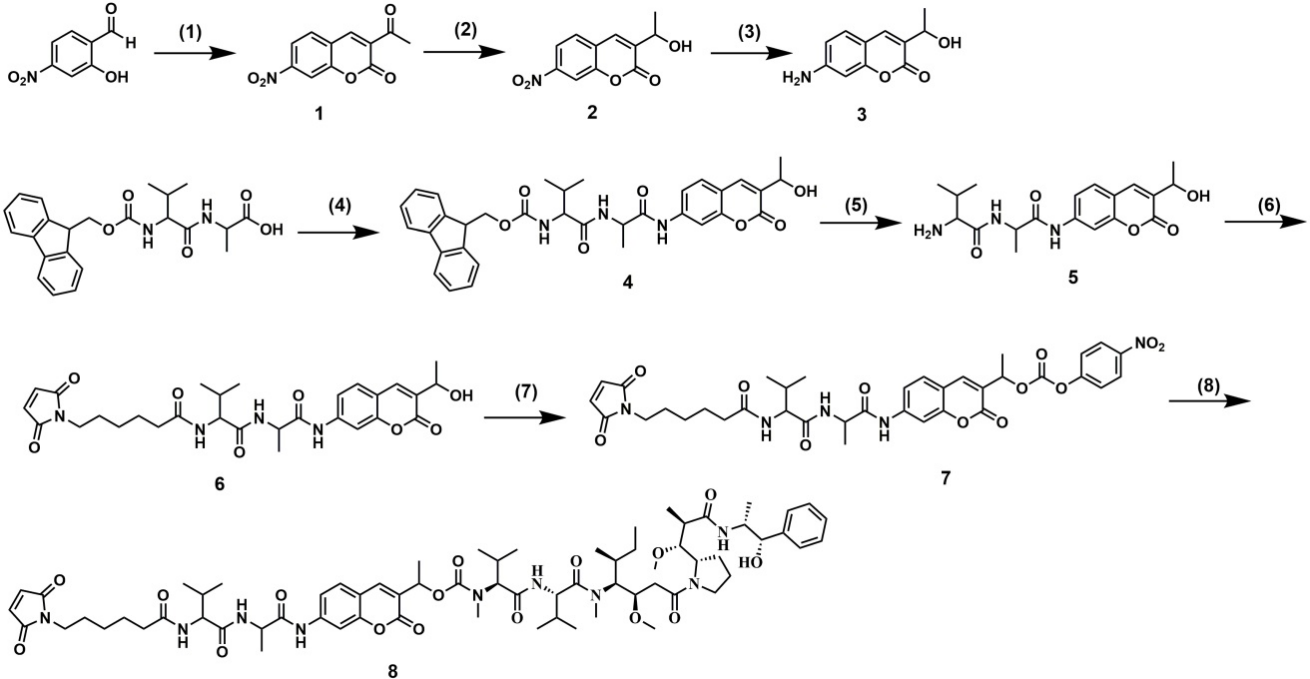
Synthesis of 7-AHC-VA-MMAE. (1) Ethyl acetoacetate, piperidine, EtOH, reflux, 1.5 h. (2) NaBH_4_, CeCl_3_, MeOH/THF = 1:1, 0 ºC, 1.5 h. (3) FeCl_3_, N_2_H_4_·H_2_O, C, EtOH, reflux, 2 h. (4) Amino coumarin, T_3_P, NMM, THF, 0 ºC, 3.5 h. (5) DMF, piperidine, RT, 1 h. (6) 6-maleimidohexanoic acid N-hydroxysuccinimide ester, DIPEA, DMF, RT, 12 h. (7) Bis(4-nitrophenyl) carbonate, DIPEA, DMF, RT, 12 h. (8) MMAE, DIPEA, HOBT, DMF, 12 h.

The synthetic route followed for the synthesis of 7-AHC-VA-MMAE (compound **8)** is shown in Figure 2. Intermediate **1** was prepared by the reaction of 2-hydroxy-4-nitrobenzaldehyde with ethyl acetoacetate in the presence of a few drops of piperidine. Then, intermediate **1** was reduced by NaBH_4_ and CeCl_3_ to obtain intermediate** 2**, which was reacted with FeCl_3_ and N_2_H_4_·H_2_O to afford key intermediate **3**. Then, intermediate **3** and Fmoc-VA were coupled with T_3_P and NMM to obtain intermediate **4**. The Fmoc protecting group was removed to provide intermediate **5**, which was then reacted with 6-maleimidohexanoic acid N-hydroxysuccinimide ester to obtain intermediate **6**. The reaction of intermediate **6** with bis(4-nitrophenyl) carbonate and diisopropylethylamine (DIPEA) resulted in a reactive intermediate that was successively reacted with MMAE to yield the desired compound **8**, 7-AHC-VA-MMAE. Detailed synthetic procedures, yields, and spectroscopic data, including ^1^H NMR, ^13^C NMR, ESI-MS, and HR-MS, are shown in Figures S10-S16.

### *In vitro* characterization of Mil40-E-15C

**Figure 3:**
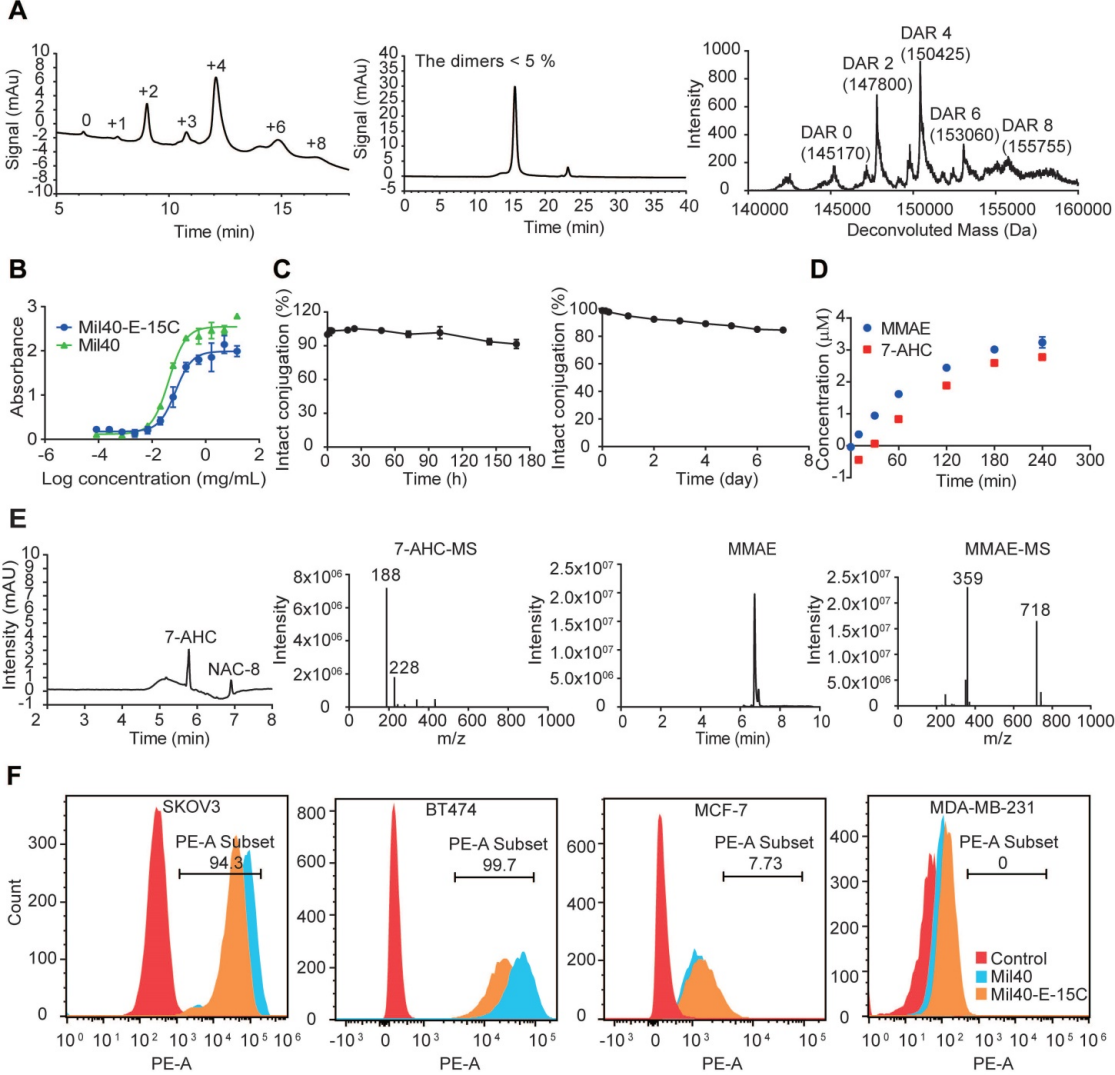
*In vitro* characterization of Mil40-E-15C. (A) HIC, polymerization degree and UPLC-Q-TOF-MS analysis of Mil40-E-15C. DAR of Mil40-E-15C = 4.2, and the dimer of the aggregated antibody peak of Mil40-E-15C was 4.65%. (B) Measuring the relative affinities of Mil40 and Mil40-E-15C to antigens of Her2. Data are presented as means ± SEM (n = 2). (C) Stability assays of Mil40-E-15C in PBS and 50% plasma at 37 °C. Data are presented as means ± SEM (n = 2 to 3). (D) *In vitro* MMAE and 7-AHC release with CTSB. Data are presented as means ± SEM (n = 3). (E) HPLC (7-AHC and NAC-**8**), SIR (MMAE), MS (7-AHC and MMAE) of NAC-**8** treated with CTSB for 4 h. (F) Mil40 and Mil40-E-15C binding to the Her2 antigens of SKOV3, BT474, MCF-7 and MDA-MB-231 cells.

Mil40-E-15C is an ADC resulting from the conjugation of MMAE to the Cys residues of anti-Her2 antibody Mil40, exerted by our novel 7-AHC-based VA dipeptide linker. Hydrophobic interaction chromatography (HIC) and UPLC-Q-TOF-MS analysis showed that Mil40-E-15C predominantly consisted of species with a drug to antibody ratio (DAR) of 2 and 4, yielding a mean DAR of 4.2 and 3.8, respectively (Figure 3A and Figure S2). The DAR values obtained with UPLC-Q-TOF-MS were slightly lower than those obtained with HIC, which might be due to the inhibition of the ionization signal by the conjugated linker payloads. According to size exclusion chromatography analysis, the ADC preparation produced a nearly exclusive (> 95% purity) single monomeric species (Figure 3A and Figure S2).

The binding capacity of the naked antibody and Mil40-E-15C to the Her2 antigen was determined using ELISA. The binding of Mil40-E-15C and Mil40 was comparable, with EC_50_ values of 0.075 μg/mL and 0.042 μg/mL, respectively (Figure 3B). Thus, the results indicated that Mil40-E-15C retains a high antigen affinity.

The stability of the novel linker was tested in phosphate-buffered saline (PBS) solution. The 7-AHC-VA-MMAE compound (compound **8**) was first added to a solution of excess N-acetyl-L-cysteine (NAC) for complete conversion to NAC-linker-MMAE (NAC-**8**) before testing. NAC-**8** was incubated in PBS at 37 °C for 7 days. Aliquots were taken at predetermined time points (0, 5, 10 and 30 min, and 1, 2, 4, 12, 24, 48, 72, 96 and 168 h) and analyzed on HPLC. The results were based on the area under the curve (AUC) of NAC-**8** at each time point as a percentage of the AUC at t = 0. After 7 days, the percentage of AUC was 90.42%, and the peak corresponding to MMAE was not observed in HPLC (Figure 3C). Then, the stability of the ADC was evaluated in human plasma. Mil40-E-15C was incubated in 50% plasma at 37 °C for 7 days. Aliquots were taken at predetermined time points (0, 3, 6, 24, 48, 72, 96 and 168 h) and analyzed with LC-MS/MS for the release of free MMAE. As shown in Figure 3C, approximately 15.52% (~ 163 nM) of the total MMAE (~ 1050 nM) in the ADC (250 nM, DAR = 4.2) was released. The above data indicated that both the novel 7-AHC-based dipeptide linker and the theranostic ADC Mil40-E-15C were highly stable.

The effective release of MMAE is very important for the efficacy of theranostic ADCs. To verify that CTSB was able to hydrolyze the novel 7-AHC-based dipeptide linker to release MMAE and consequently activate 7-AHC, an enzymatic hydrolysis experiment was performed with NAC-**8**. The released MMAE and 7-AHC were monitored with LC-MS. As shown in Figure 3D and Figure 3E, MMAE was released smoothly over time, accompanied by the generation of an almost equimolar amount of 7-AHC. These results indicated that the cleavage of the 7-AHC-based dipeptide linker by CTSB generates a novel fluorescence on-off sensor, 7-AHC, which undergoes spontaneous 1, 6-elimination to release the active payload, MMAE.

The affinity of theranostic ADC Mil40-E-15C and unconjugated antibody Mil40 was further compared in Her2+ (BT474 and SKOV3) and Her2- (MCF-7 and MDA-MB-231) cells (Figure 3F). The mean PE fluorescence intensity of Mil40-E-15C was slightly lower than that of Mil40 in BT474 and SKOV3 cells (26429 *vs.* 57304, and 42628 *vs.* 75660, respectively). Both Mil40-E-15C and Mil40 showed weak PE fluorescence in MCF-7 and MDA-MB-231 cells. These results further demonstrated that Mil40-E-15C specifically targeted the Her2 antigen of tumor cells.

### Fluorescence on-off properties

**Figure 4:**
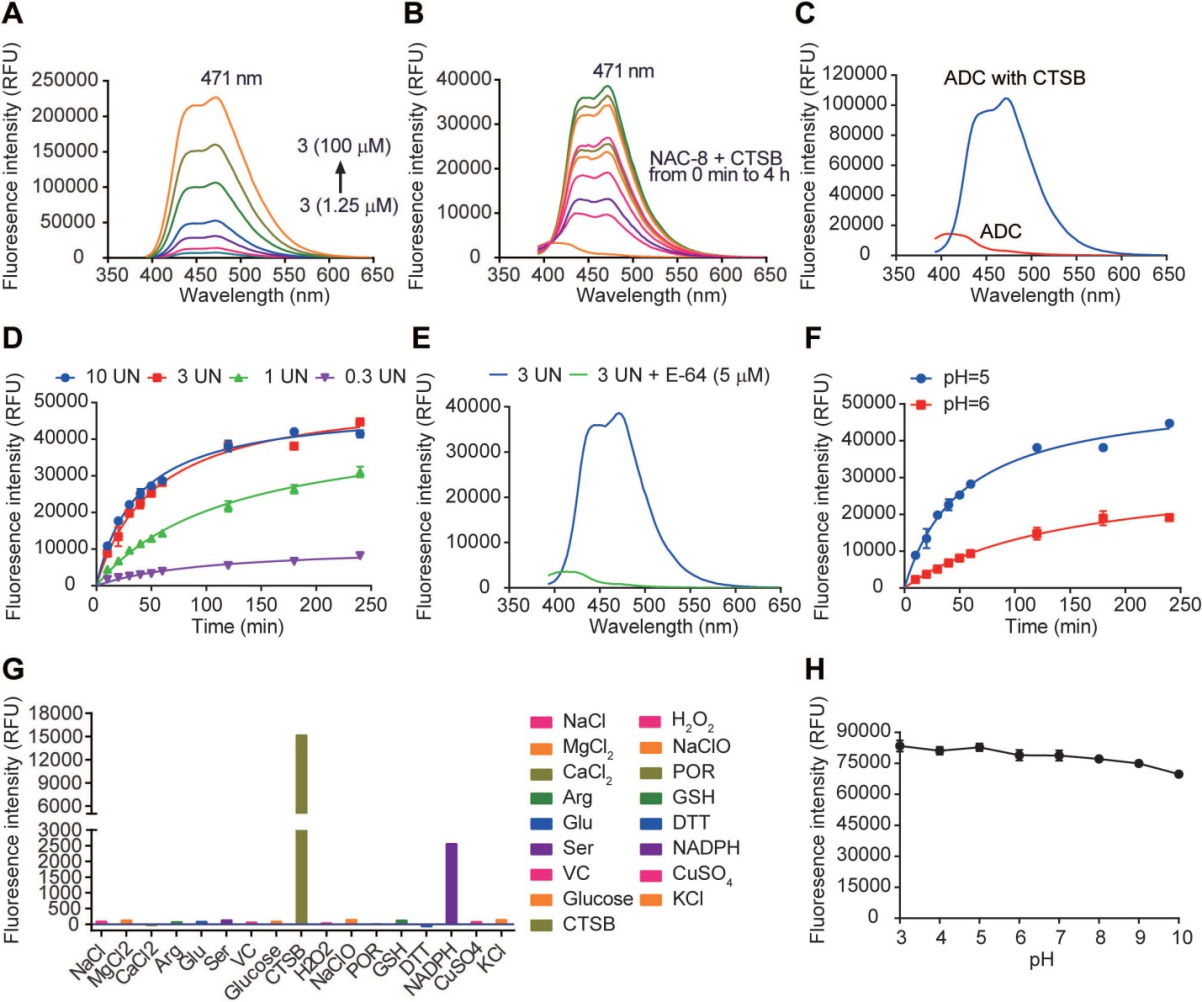
Fluorescence properties of 7-AHC, 7-AHC based linker NAC-**8** and 7-AHC based ADC Mil40-E-15C. (A) Fluorescence spectra of 7-AHC (1.25, 2.5, 5, 10, 25, 50, 100 µM). (B) The fluorescence response of 10 µM NAC-**8** with CTSB. (C) The fluorescence response of 10 µM Mil40-E-15C with and without CTSB. (D) Changes in fluorescence intensity at 471 nm as a function of CTSB concentration. Data are presented as means ± SEM (n = 2). (E) Changes in fluorescence intensity at 471 nm as a function of pH. Data are presented as means ± SEM (n = 2). (F) The fluorescence response of 10 µM NAC-**8** with CTSB in the presence of CTSB inhibitor E-64. (G) Changes in fluorescence intensity at 471 nm in the presence of various species. (H) Changes in fluorescence intensity of 10 µM 7-AHC as a function of pH. Data are presented as means ± SEM (n = 2). All spectra were acquired 4 h after addition of the analyte at 37^o^C in PBS buffer (pH 7.4, except for indicated) containing 10% DMSO (v/v) with excitation at 371 nm.

As a new fluorescence on-off sensor, the fluorescence properties of 7-AHC needed to be clarified. First, the absorption spectrum (Figure S3) and fluorescence emission spectrum (Figure 4A) of 7-AHC were monitored with UV-vis and fluorescence spectroscopy. In the UV spectrum, 7-AHC showed a strong absorption peak at λ_max_ = 346 nm and exhibited strong fluorescence centered at 471 nm. The fluorescence of NAC-**8** increased in intensity by approximately 48-fold within 1 h of treatment with CTSB (3 UN) in PBS buffer (pH 7.4) containing 10% DMSO (v/v). The fluorescence spectra of the NAC-**8** solution after the addition of CTSB were exactly the same as the emission spectrum of the 7-AHC reference (Figure 4B). The results preliminarily indicated that 7-AHC is a novel self-elimination fluorescence on-off sensor. The fluorescence of Mil40-E-15C with or without CTSB was also examined and shown to increase from 3101 to 104603 upon treatment with CTSB (Figure 4C).

Furthermore, fluorescence at λ_max_ = 471 nm was monitored in the presence of different concentrations of CTSB over time. The fluorescence intensity of NAC-**8** at λ_max_ = 471 nm increased until reaching a saturation point at 3 UN CTSB (Figure 4D). Moreover, the initial rates of fluorescence intensity with time were largely dependent on CTSB concentration, and as shown in Figure 4D, V_max_ was 10958, 45257 and 52928 at 0.3 UN, 1 UN and 3 UN CTSB, respectively. In contrast, after pre-treatment with the CTSB inhibitor E-64 (5 μM) for 15 min, no significant fluorescence enhancement at λ_max_ = 471 nm was observed at 3 UN CTSB (Figure 4E). These results showed that the cleavage of the novel 7-AHC-based dipeptide linker by CTSB occurs in a dose-dependent manner.

Another set of experiments was performed to determine the role of pH in triggering the release of NAC-**8** (10 μM). In the presence of CTSB, the fluorescence intensity increased faster at pH 5 than at pH 6 (V_max_ = 52928 and 32185, respectively, Figure 4F). These results indicated that CTSB works more effectively under acidic conditions, which are similar to those of the acidic microenvironment of lysosomes.

Before *in vitro* testing, the selectivity of NAC-**8** for CTSB over other biological species was evaluated. To assess the possibility of interference, NAC-**8** was reacted with various biological species, including NaCl, MgCl_2_, CaCl_2_, Arg, Glu, Ser, VC, glucose, H_2_O_2_, NaClO, POR, GSH, DTT, NADPH, CuSO_4_, KCl and CTSB. As seen in Figure 4G, increased fluorescence intensity was only observed when NAC-**8** was reacted with CTSB. No significant changes in fluorescence intensity were observed after the addition of other biological species. These findings indicated that NAC-**8** selectively reacts with CTSB. Additionally, it seems that the fluorescence of 7-AHC decreased slightly under alkaline conditions (Figure 4H). Overall, it could be concluded that the pH had little impact on the fluorescence of 7-AHC.

Compared with the traditional methods for monitoring drug release, such as complex mass spectrometry-based techniques 26, drug release with our novel 7-AHC-based linker could be directly monitored using ELISA due to the fluorescence characteristics of 7-AHC.

### Cytotoxicity of Mil40-E-15C *in vitro*

**Figure 5:**
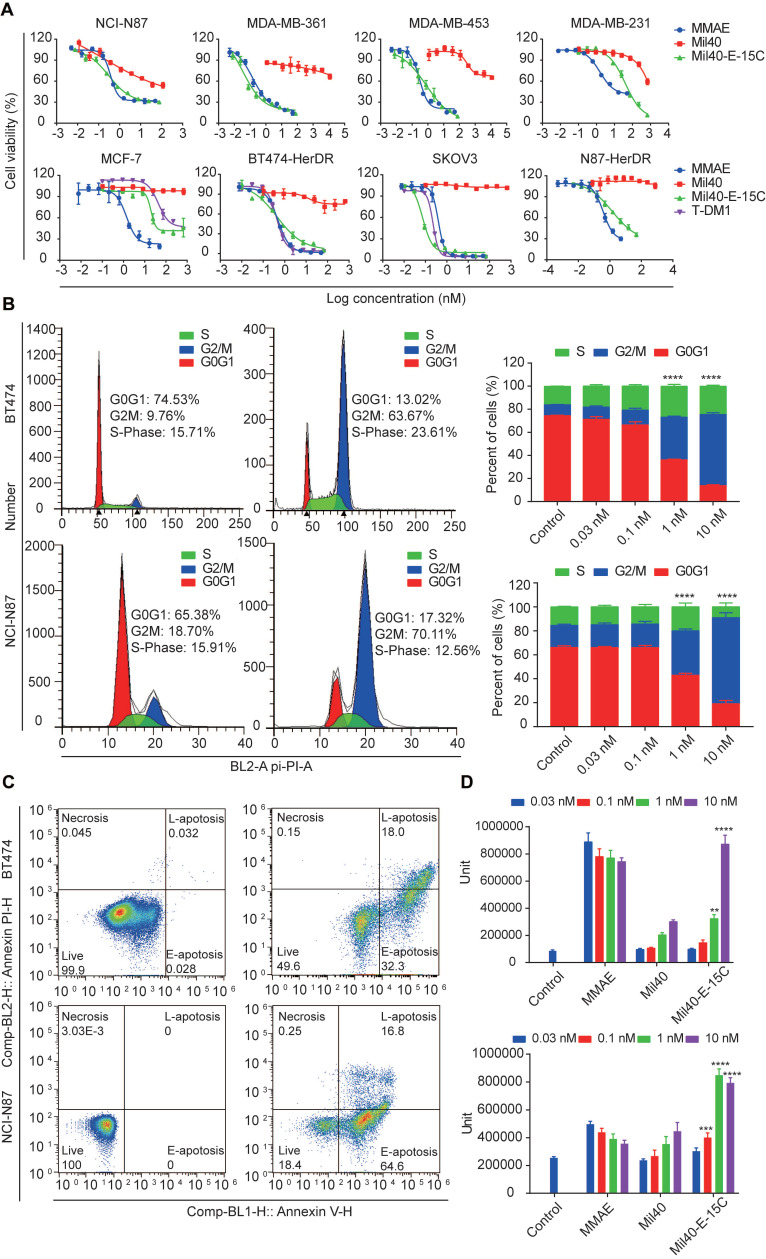
Cytotoxicity of the theranostic ADC in tumor cells. (A) The cytotoxicity assays were performed in Herceptin-sensitive and Herceptin-resistant cell lines. Data are presented as means ± SEM (n = 2 to 3), IC_50_ values were calculated from a 4-variable curve analysis by OriginPro 2018. (B) Cell-cycle analysis of Mil40-E-15C under the concentration of 0 nM and 10 nM in BT474 and NCI-N87 cells. Data are presented as means ± SEM (n = 3). To compare the difference in activity between control and ADC, two-way ANOVA were used, and statistical analyses were performed using Prism 7.0. ****p < 0.0001. (C) Apoptosis analysis of Mil40-E-15C under the concentration of 0 nM and 10 nM in BT474 and NCI-N87 cells. (D) Caspase-3/7 activity analysis of Mil40-E-15C under the concentration of 0 nM, 0.03 nM, 0.1 nM, 1 nM, 10 nM in BT474 and NCI-N87 cells. Data are presented as means ± SEM (n = 3). To compare the difference in activity between antibody and ADC, two-way ANOVA were used, and statistical analyses were performed using Prism 7.0. **p < 0.005, ****p < 0.0001.

Herceptin-sensitive cell lines were selected on the basis of their published Her2 status, including Her2+ (gastric carcinoma NCI-N87 and breast carcinoma MDA-MB-361 and MDA-MB-453), and Her2- (breast carcinoma MCF7 and MDA-MB-231) cells. The Her2 status of these cell lines was confirmed with flow cytometric (Figure S4). The relative MFIs of NCI-N87, MDA-MB-361, MDA-MB-453, MCF-7 and MAD-MB-231 cells were 272.01, 41.14, 29.81, 3.39 and 2.16, respectively. Cytotoxicity studies in all cell lines confirmed high sensitivity to MMAE with potencies of 0.2 nM to 1.93 nM (Table S1, Figure 5A). Mil40-E-15C demonstrated potent inhibitory activities in the NCI-N87, MDA-MB-361 and MDA-MB-453 Her2+ cell lines, with 50% inhibition concentration (IC_50_) values of 0.74 nM, 0.11 nM, and 1.22 nM, respectively (Table S1, Figure 5A). In addition, we confirmed the relatively weak inhibitory activity of Mil40. Moreover, Mil40-E-15C exhibited low activity against Her2- cell lines (IC_50_ = 32.48 and 15.18 nM). Thus, the cell growth inhibitory activity of the theranostic ADC was confirmed to be Her2-specific.

Next, Mil40-E-15C was evaluated for cytotoxicity against trastuzumab-resistant cell lines (BT474-HDR, SKOV3, and N87-HDR). The relative MFIs of BT474-HDR, SKOV3 and N87-HDR cells were 157.45, 392.53 and 136.45, respectively, suggesting that Her2 was clearly expressed on their cell surface (Figure S5); additionally, the IC_50_ values were 0.58 nM, 0.09 nM and 3.74 nM (Table S1, Figure 5A), respectively. For comparison, the clinically approved ADC T-DM1 was also evaluated for its cytotoxicity against BT474-HDR, SKOV3 and MCF-7, demonstrating similar potencies. The IC_50_ values were 0.46 nM, 0.24 nM and 184.82 nM, respectively (Table S1). As a control, Mil40 exhibited no activity against the trastuzumab-resistant cell lines (IC_50_ > 100 nM). These results indicated that the new theranostic ADC maintains the high activity advantage of the clinically approved ADC.

MMAE impairs tubulin polymerization, causing cell cycle arrest in the G2/M phase and eventually leading to apoptosis 31 (Figure S6 and S7). MMAE could induce robust cell cycle arrest at very low concentrations (0.03 nM). The percentage of cells in the G2/M stage increased to 77.84% (Figure S6). As shown in Figure 5B, Mil40-E-15C induced G2/M-phase arrest in target cells preceding the onset of apoptosis. After treatment with Mil40-E-15C for 24 h at concentrations of 0.03 nM, 0.1 nM, 1 nM and 10 nM, the percentages of BT474 cells in the G2/M phase were 10.60%, 12.95%, 37.06% and 61.68%, respectively. In contrast, after treatment with Mil40 at the same concentrations, the percentages of BT474 cells in the G2/M phase were 9.68%, 8.16%, 8.70% and 8.12%, respectively. To examine apoptosis, BT474 cells were treated for another 24 h, and the percentage of cellular apoptosis was 17.0%, 33.7%, 48.1% and 81.4% at the concentrations of 0.03 nM, 0.1 nM, 1 nM and 10 nM, respectively (Figure 5C, Table S2). Meanwhile, Mil40 only had a limited impact on cell apoptosis (Figure S6 and S7). Similar trends were also observed in N87 cells (Table S2). These results suggested that the theranostic ADC exerts cytotoxicity through the release of MMAE, which impairs tubulin polymerization. Moreover, caspase-3/7 activity was determined for an accurate analysis of apoptosis (Figure 5D). Mil40-E-15C induced BT474 and NCI-N87 cell apoptosis in a dose-dependent manner. In addition, the percentage of apoptosis induced by Mil40-E-15C was significantly higher (*p* < 0.005) than that induced by Mil40 at the same concentration. These results further verified that the apoptotic activity was induced by the payload MMAE.

### Imaging of payload release

**Figure 6:**
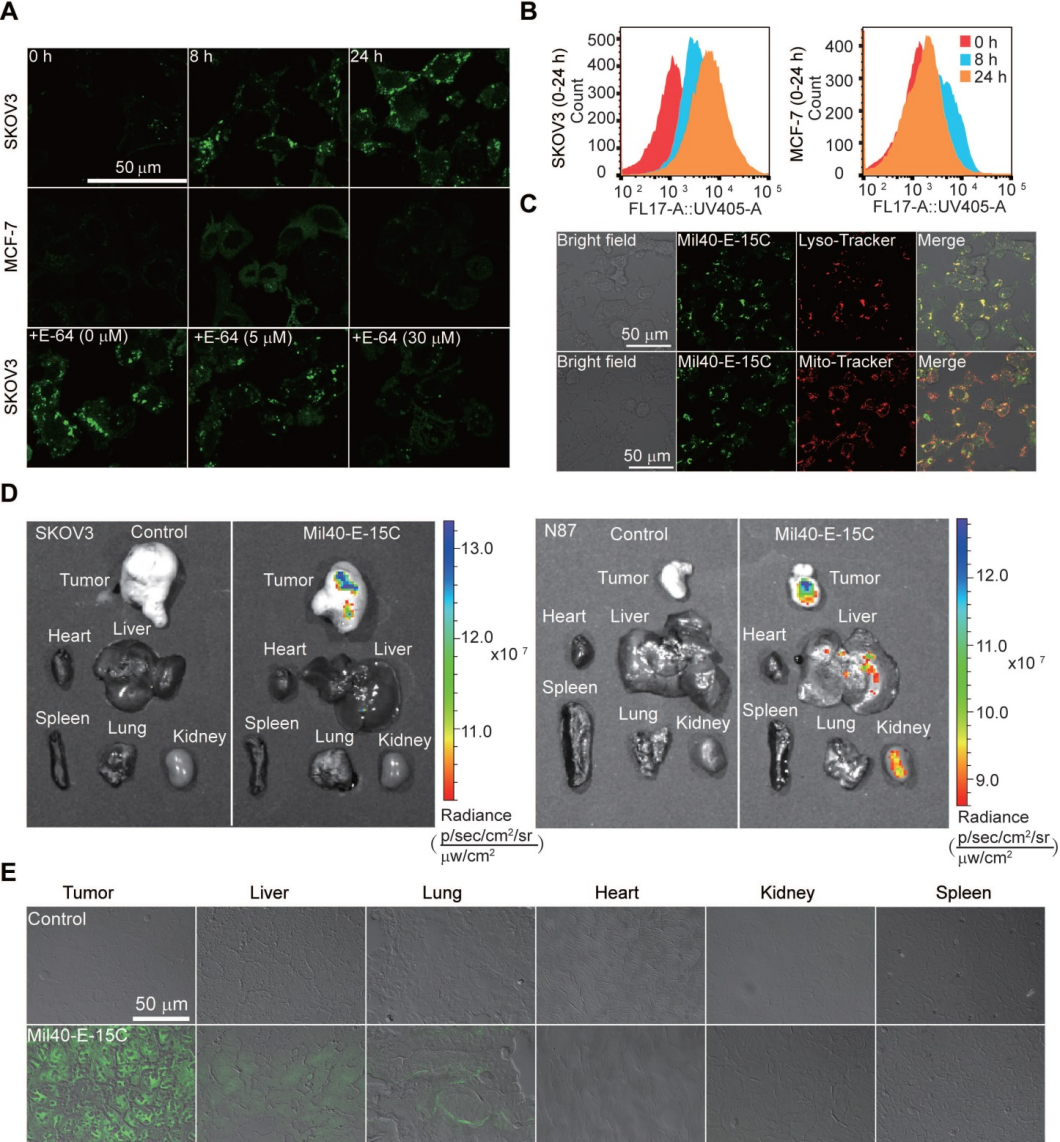
The drug release was demonstrated by flow cytometric analysis and confocal microscope. (A) The live-cell imaging of SKOV3 and MCF-7 cells after 0 h, 8 h or 24 h of ADC treatment revealed drug releasing at 1 mg/mL Mil40-E-15C. Excitation at 405 nm for Mil40-E-15C (Green). The live-cell imaging of SKOV3 cells pretreated with cathepsin inhibitor E-64 for 8 h, was obtained after 24 h of Mil40-E-15C treatment. Scale bars: 50 µm. (B) For flow cytometric analysis, Mil40-E-15C was excited at 365 nm and the emission was monitored at 505 ± 15 nm. (C) Subcellular localization of Mil40-E-15C in SKOV3 cells. Cells were treated with 1 mg/mL Mil40-E-15C for 24 h. Then LysoTracker-Red was used to identify the lysosomes and MitoTracker-Red was used to identify mitochondria. Scale bars: 50 µm. (D) Fluorescence images of tumor region and major organs obtained from SKOV3/NCI-N87-cell-inoculated xenograft mice after administration with Mil40-E-15C and PBS. Exposure time: 60 s. Excitation at 430 nm (emission channel = 500 nm) for Mil40-E-15C. (E) Confocal microscopy images of the tissue sections taken from a SKOV3-cell-inoculated xenograft mice that were injected with Mil40-E-15C (100 mg/kg in L-Histidine buffer solution) and L-Histidine buffer solution, respectively. Each panel shows an overlay of the fluorescence image with bright field one. Scale bars: 50 µm.

With the theranostic ADC in hand, we could conveniently visualize intracellular drug release using confocal microscopy and flow cytometry. Time-course imaging experiments showed that the ADC released the payload after incubation for 8 h in SKOV3 cells (Figure 6A). Simultaneously, an increase in fluorescence was observed in the cells. Moreover, intracellular fluorescence was further enhanced after 24 h of incubation. In contrast, MCF7 Her2- cells showed little to no fluorescence signal under similar experimental conditions. To provide further evidence for CTSB-induced dipeptide bond cleavage as well as concomitant fluorescence, Mil40-E-15C was studied in the presence of the CTSB inhibitor E-64. The fluorescence intensity of Mil40-E-15C in SKOV3 cells decreased with increasing concentration of E-64 (0-30 μM) (Figure 6A). This result indicated that Mil40-E-15C could enter the cells through receptor-mediated endocytosis and that the fluorescence came from the 7-AHC generated by the dipeptide linker cleavage of Mil40-E-15C under the action of intracellular CTSB.

To quantify intracellular drug release, we analyzed our theranostic ADC with flow cytometry. Previously, radioisotopic labeling of the payload and its extraction from the cell with organic solvents were needed 32-34. Herein, we could assess the intracellular processing of our theranostic ADC in live cells using flow cytometry. In the assay, the mean fluorescence intensity after treatment of SKOV3 cells with Mil40-E-15C was 1319, 4251 and 8705 at 0, 8 and 24 h, respectively (Figure 6B). However, that on MCF7 remained unchanged (Figure 6B). These results were consistent with the confocal microscopy results; thus, both sets of results provide evidence for the intracellular release of MMAE and 7-AHC.

Since CTSB is widely distributed in lysosomes, previous studies have reported that the release of drugs in ADCs mainly occurs in these compartments 35; however, direct evidence is still lacking. To detect the intracellular drug release of theranostic ADCs, the localization of activated theranostic ADCs triggered by CTSB was investigated using fluorescent markers for lysosomes (LysoTracker-Red) and mitochondria (MitoTrackerRed) (Figure 6C and S8). As shown in Figure 6C, the fluorescence signal of Mil40-E-15C colocalized with LysoTracker (Pearson's coefficient = 0.83), while only a slight colocalization with MitoTracker was observed. These results provided direct evidence that ADC drug release occurred in the lysosomes.

To examine drug release and the distribution of MMAE in tumors and various organs, *ex vivo* imaging was performed by monitoring the fluorescence emission. SKOV3/NCI-N87-cell-inoculated xenograft mice were administered Mil40-E-15C via tail vein injection. The tumor-specific accumulation of Mil40-E-15C indicated its target specificity as visualized by the enhanced fluorescence emission in the solid tumor (Figure 6D) compared with that in the control tissue. Liver and kidney tissues also showed enhanced fluorescence signals because of the metabolization and excretion of Mil40-E-15C. However, weak or no fluorescence was observed in the lung, spleen, and heart. To further validate the fluorescence characteristics of Mil40-E-15C, fluorescence images of various tissue sections (thickness: 10 μm) were obtained using confocal microscopy. Figure 6E shows the fluorescent images of Mil40-E-15C in the tumor, liver, lungs, heart, and spleen. The images confirmed that Mil40-E-15C accumulated selectively in the tumor and liver, as reflected in observable fluorescent imaging, whereas little fluorescence was detected in other organs. On the basis of the studies detailed above, we inferred that this occurred as a result of antibody-based targeting and Her2 receptor-mediated endocytosis, and that once taken up into tumor cells, CTSB-induced dipeptide bond cleavage in Mil40-E-15C to release the MMAE payload and 7-AHC.

### *In vivo* potency in xenografted mice

**Figure 7:**
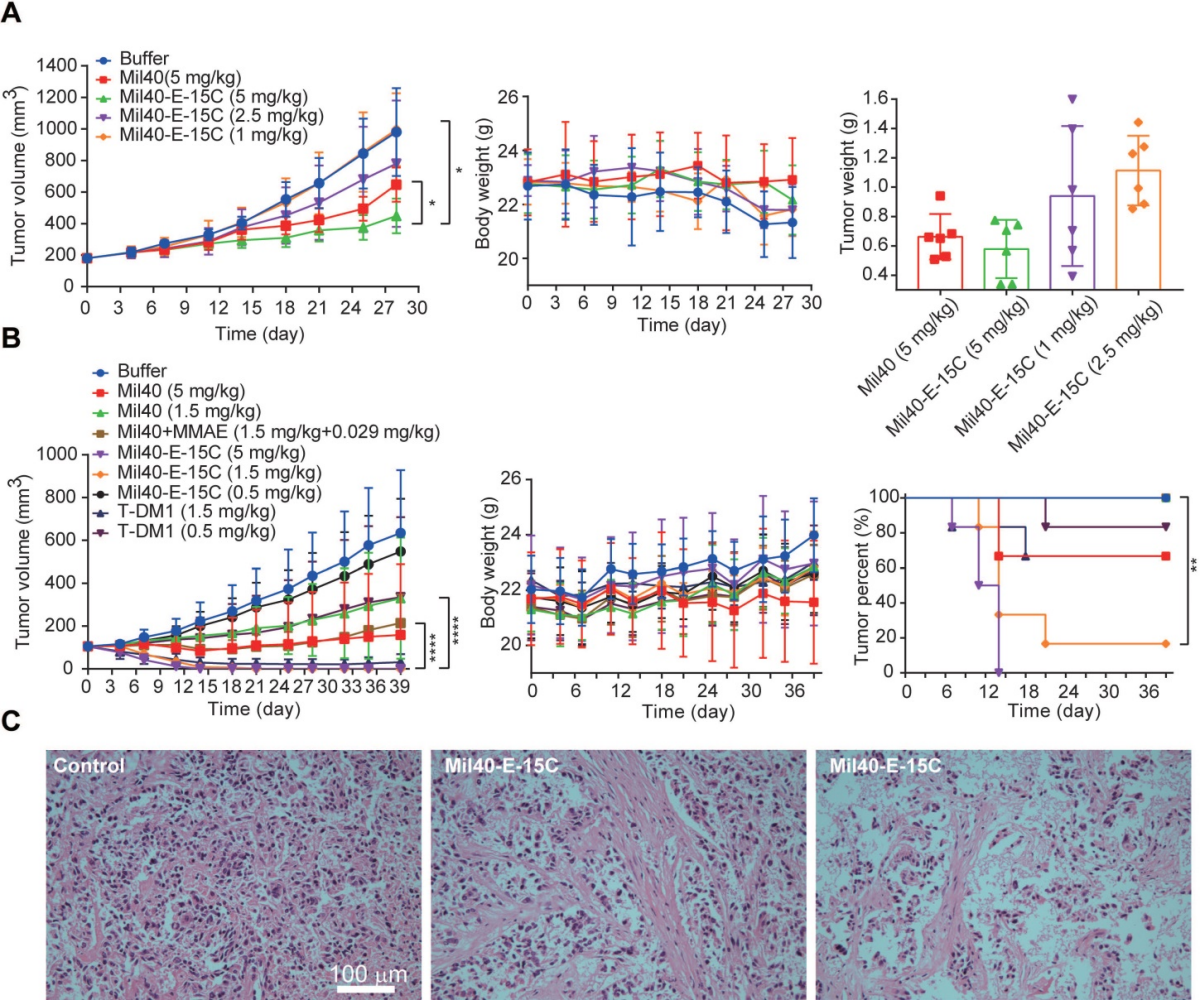
* In vivo* effects of Mil40-E-15C in xenografted mice. (A) NOD/SCID female mice were implanted subcutaneously with SKOV3 cells. When the tumors reached ~ 180 mm^3^, the mice were given vehicle, Mil40, and ADC on days 0, 7, 14 and 21 respectively. Results are shown as mean ± SD, n = 6/group. The tumor volume, changes in body weight and tumor weight of the test mice during the treatment and observation period. To compare the difference in activity between Mil40 and ADC, unpaired two-tailed t tests were used, and statistical analyses were performed using Prism 7.0. *p < 0.05. (B) CB17.SCID female mice were implanted subcutaneously with BT474 cells. When the tumors reached ~ 100 mm^3^, the mice were given vehicle, Mil40, and ADC on days 0, 7, 14 and 21 respectively. Results are shown as mean ± SD, n = 6/group. The tumor volume, changes in body weight and tumor disappearance time of the test mice during the treatment and observation period. To compare the difference in activity between antibody, combined treatment and ADC, unpaired two-tailed t tests or survival analyses were used, and statistical analyses were performed using Prism 7.0. ****p < 0.0001, **p < 0.005. (C) Histological sections of SKOV3 tumor tissues with H&E staining. Scale bars: 100 µm.

Finally, to evaluate the anticancer efficacy of Mil40-E-15C *in vivo,* we investigated its therapeutic potential in SKOV3 and BT474 cell-inoculated xenograft mice (Figure 7A-C). In our recent study 36, we monitored the half-life of fluorescence-labeled Mil40 in nude mice using small animal imaging technology. The results indicated a half-life of 5-7 days. Therefore, we chose a weekly administration scheme during the *in vivo* study. As shown in Figure 7A, SKOV3 cell-inoculated xenograft NOD-SCID mice were intravenously administered Mil40-E-15C at doses of 1, 2.5 and 5 mg/kg or Mil40 at a dose of 5 mg/kg on days 0, 7, 14 and 21. Compared with the control group, the 5 mg/kg ADC treatment group produced significant and sustained antitumor effects with a 54.3% tumor inhibition rate (Figure 7A). However, no significant tumor suppression (*p* > 0.05) was observed in the group treated with 5 mg/kg Mil40 when compared with the control group. This indicated that the ADC had a significant increase in potency compared with Mil40 (*p* < 0.05). In addition, the tumor weight of the Mil40-E-15C group was lower than that of the Mil40 group. During the treatment period, no weight loss was observed in any of the treatment groups. The body weights of the test mice were not significantly different between the experimental and control groups, indicating that the ADC and naked antibody were preliminarily well tolerated at the therapeutic doses used (Figure 7A). The histological effect of the ADC on tumors was investigated using hematoxylin and eosin (H&E) staining (Figure 7C). Clear fibrosis and necrosis of tumor tissue were observed in the ADC treatment group. The results showed that the new theranostic ADC possessed a strong anticancer effect in mice, thus preliminarily achieving our design purpose.

To further clarify the *in vivo* efficacy of theranostic ADC, we conducted a well-controlled study in the classic breast cancer model. As shown in Figure 7B, BT474 cell-inoculated xenograft CB17.SCID mice were intravenously administered all the testing drugs once a week, a total of four times. The controls included a Mil40-treated group, a Mil40 combined with MMAE-treated group, and a group treated with the clinically approved ADC T-DM1. Mil40-E-15C exhibited a striking antitumor effect. First, Mil40-E-15C showed significant stronger antitumor effect (*p* < 0.0001) than the Mil40 and combination groups at the same dose (Figure 7B and Figure S9A-B). More importantly, two doses of 5 mg/kg Mil40-E-15C induced tumor regression in all six mice, and four doses of 1.5 mg/kg Mil40-E-15C induced tumor regression in five out of six mice. In contrast, four doses of 1.5 mg/kg T-DM1 induced tumor regression in only two mice (Figure 7B and Figure S9A-B). By comprehensive comparison, the theranostic ADC exhibited the equivalent antitumor efficacy to the marketed ADC T-DM1. No significant body weight changes were found in any of the groups during the treatment, which indicated the safety of Mil40-E-15C. Good* in vivo* efficacy might be another important bright spot of theranostic ADC. Besides, in immunocompromised mice lacking T and B cells, the immune killing function of antibodies might be affected to some extent. Although it is generally accepted that targeting might be the most important function of the antibody in ADCs, the tumor-killing effect of ADCs mainly depends on the highly toxic payload.

## Conclusion

In conclusion, we report here a new theranostic ADC, Mil40-E-15, which contains a novel bifunctional molecule consisting of 7-AHC as a fluorescent signal and self-elimination moiety. Based on an “all in one” design concept, we constructed this ADC with Her2 antibody Mil40 for effective group targeting; thus, our new 7-AHC-based dipeptide linker contained an on-off fluorescence linker and MMAE as an active payload. The viability of the design was tested under controlled conditions. For instance, the 7-AHC-based dipeptide linker exhibited similar payload release properties as a PAB-containing linker. An almost equimolar amount of 7-AHC was generated when MMAE was released. As designed, the 7-AHC-based dipeptide linker gave rise to almost no fluorescence signal. However, exposure to CTSB, but not a number of potential interferants, led to an approximately 48-fold increase in fluorescence intensity. Therefore, *in vitro* drug release could be conveniently monitored owing to this fluorescence characteristic. In cells treated with Mil40-E-15C, an enhancement in fluorescence intensity over time was correlated with the release of 7-AHC and MMAE. Direct evidence for drug release in lysosomes was provided in the colocalization analysis. Thus, it was concluded that the fluorescence of 7-AHC may be used to conveniently track intracellular colocation and CTSB-induced release of MMAE from the theranostic ADC. In addition, Mil40-E-15C showed strong cell inhibitory activity in Her2+ cells, with IC_50_ = 0.09-3.74 nM. Flow cytometry analysis demonstrated that it exerts antitumor effects via tubulin polymerization impairment, which subsequently leads to cell apoptosis. Moreover, *ex vivo* fluorescence imaging analyses, carried out after intravenous injection of Mil40-E-15C into mice containing SKOV3 cell xenografts, confirmed the enhanced fluorescence emission in the solid tumor and liver tissues. These results indicated the remarkable tumor targeting capacity of Mil40-E-15C. More importantly, this ADC also had a significant increase in potency (p < 0.05) compared with Mil40 in SKOV3 and BT474 tumor models. The most exciting finding was that the antitumor effect of Mil40-E-15C was comparable to that of marketed ADC T-DM1 in the classic breast cancer model. On this basis, we concluded that Mil40-E-15C represents a potentially useful new approach to theranostic design, which can provide new tools for intracellular payload release. To our knowledge, this is the first report of a universal strategy, applicable in all PAB-containing ADCs, that can promote the preclinical study of PAB-containing ADCs and the development of theranostic systems.

## Experimental Section

### Synthesis information

For details, see supplementary Methods.

### Bioconjugation and purification

Humanized anti-Her2 IgG1 Mil40 antibodies (10 mg/mL) in L-histidine buffer (20 mM), pH 7.2, were treated with tris(2-carboxyethyl)phosphine hydrochloride (TCEP; 2.3 equivalents) at 25 °C for 90 min. The compound **8** (8 equivalents) was added to the reduced Mil40 in ice-cold dimethylacetamide (DMAC) (8% v/v). After 120 min, the reactions were quenched with excess NAC (10 equivalents). The mixture was placed on ice for 30 min and then eluted through Sephadex-G25S for buffer exchange. The conjugates were sterile-filtered through a 0.2 μm filter under sterile conditions and stored at -80 °C before use for analysis and testing. The clinically approved ADC T-DM1 was purchased from ShangHai Biochempartner Co., LIMITED.

### Quality control

The DAR value of Mil40-E-15C were determined with hydrophobic interaction chromatography, UPLC-Q-TOF-MS and the polymerization degree were determined with size exclusion chromatography. The capacity of Mil40-E-15C binding to the Her2 antigen was analyzed with ELISA. Her2 antigen (100 μL, 1 μg/mL) in the coating solution was plated on each well of a 96-well plate, which was placed at 4 °C overnight in a wet box. The protein-binding sites of each well were blocked by incubation with 10% fetal bovine serum for 2 h at 37 °C. Each well was treated with Mil40-E-15C or Mil40 with gradient concentration at 37 °C for 1 h and washed three times with PBS containing 0.2% Tween 20. Then, 100 μL of goat anti-human IgG Fc cross-adsorbed secondary antibody conjugating HRP (Thermo Fisher Scientific, USA) was added into each well and incubated for 45 min at room temperature. The plated cells were washed three times with PBS containing 0.2% Tween 20. TMB reagent was added to the wells to develop color, followed by the addition of 2M H_2_SO_4_ to stop the color-development process. Finally, the absorbance of each well was measured at 450 nm using a microplate reader (VersaMax, Molecular Devices, Sunnyvale, CA).

### Stability and payload release

A stock solution of NAC-**8** in PBS was incubated at 37 °C for a period of 7 days. The remaining NAC-**8** was monitored on HPLC at various time-points. To determine the stability of Mil40-E-15C in human plasma, MMAE was diluted into 2 × diluted human plasma to prepare the standard sample, and Mil40-E-15C sample preparation was the same as standards. Both samples were incubated at 37 °C for a period of 7 days. The concentration of total free MMAE released from Mil40-E-15C was determined at various time-points according to the established MMAE standard curve using LC-MS.

Stock solutions of NAC-**8** were prepared in PBS buffer containing 50% (v/v) DMSO. Next, 5 μL of NAC-**8** was added to 10 μL CTSB (15 UN) in CTSB activity buffer (50 mM sodium acetate, 100 mM NaCl, 8 mM L-cysteine, and 1 mM EDTA, pH 5.0) and 35 μL CTSB activity buffer. Aliquots (50 μL) were taken at subsequent time points (t = 0, 10, 30, 60, 80, 120, 180 and 240 min). Then, 250 μL methanol was added and centrifugation was carried out for 10 minutes for antibody sedimentation. Released MMAE and 7-AHC were analyzed on LC-MS and quantified using the standard curve method.

### Spectroscopic materials and methods

Stock solutions of biologically relevant analytes (NaCl, MgCl_2_, CaCl_2_, Arg, Glu, Ser, VC, Glucose, CTSB, H_2_O_2_, NaClO, POR, GSH, DTT, NADPH, CuSO_4_ and KCl) were prepared in PBS buffer. NAC-**8** was prepared by the reaction of compound **8** with excessive NAC. Stock solutions of NAC-**8** and 7-AHC were prepared in PBS buffer containing 50% (v/v) DMSO. A stock solution of the CTSB inhibitor E-64 was prepared in triple distilled water. Stock solutions of CTSB (Sigma) with different concentrations were prepared in CTSB activity buffer (50 mM sodium acetate, 100 mM NaCl, 8 mM L-cysteine, 1 mM EDTA, pH 5.0 or 6.0). All spectroscopic measurements were performed in a simulated lysosomal environment (Cathepsin B activity buffer containing 10% (v/v) DMSO, pH 5.0 or 6.0, 37 °C). Both absorption and fluorescence spectra were recorded using a microplate reader. Samples for absorption and emission measurements were obtained from wells of 96-well plates. Excitation was carried out at 373 nm.

### *In vitro* cell viability assay

The *in vitro* potency of Mil40, Mil40-E-15C, and free drug MMAE was assessed in cell viability assays. NCI-N87 (2.5 × 10^3^ cells/well), BT474 (2.5 × 10^3^ cells/well), MDA-MB-361 (2.5 × 10^3^ cells/well), MDA-MB-453 (2.5 × 10^3^ cells/well), MCF7 (1.5 × 10^4^ cells/well), MDA-MB-231 (1.5 × 10^4^ cells/well), SKOV3 (2.5 × 10^3^ cells/well), NCI-N87-HDR (2.5 × 10^3^ cells/well) and BT474-HDR (2.5 × 10^3^ cells/well) cells were grown in 96-well plates and treated with Mil40, Mil40-E-15C, MMAE, and T-DM1. The plate was incubated for 4 days at 37 °C, 5% CO_2_, and 95% humidity. Then, the cytotoxicity of the samples was established using the CellTiter-Glo assay kit (CTG). IC_50_ values were calculated from a 4-variable curve analysis by OriginPro 2018.

### Cell cycle arrest and apoptosis analysis

To evaluate cell apoptosis in various types of cells, Her2-positive cell lines (BT474 and NCI-N87) were seeded at a density of 2 × 10^6^ cells/well in 12-well plates and exposed to Mil40, Mil40-E-15C, and MMAE at various concentrations (0.033, 0.1, 1 and 10 nM) for 24 h. Apoptosis and cell death were detected using an Annexin V-FITC Apoptosis Kit (Dojindo, Japan) and propidium iodide (PI) staining with FACSCalibur (BD Biosciences). For cell cycle position analysis after drug exposure, the cells were treated as described above and allowed to incorporate bromodeoxyuridine (BrdU; Beyotime Biotechnology, Shanghai, China) for 20 min. Nascent DNA synthesis was detected with anti-BrdUrd FITC, and total DNA content was detected with PI. Cell cycle position and apoptosis analyses were measured using FACSCalibur (BD Biosciences).

To detect the activity of Caspase3/7, Her2-positive cell lines (BT474 and NCI-N87) were seeded at a density of 6 × 10^3^ cells/well in 96-well plates and exposed to the drugs abovementioned. After incubation for 48 h, 100 μL Caspase3/7 reagents were added to each well. The plates were then placed on a horizontal shaker and incubated at room temperature for 1 h. The chemiluminescence values of each well were read using a TECAN spark 10M multifunctional enzyme marker.

### *In vitro* uptake assay

Cultured SKOV3, and MCF7 cells were seeded in 12-well Costar® plates (4 × 10^4^ cells/well) and incubated for 24 h. The cells were then exposed to Mil40-E-15C, which was diluted in DMEM at a final dose of 1 mg/mL for 8 and 24 h at 37 °C. To quantify the cellular uptake of Mil40-E-15C, the test samples were aspirated, and the cells were trypsinized with trypsin/EDTA, washed twice with PBS, and resuspended in 1 mL of PBS, after which flow cytometric analysis (Cytomics FC 500, Beckman Coulter, USA) was performed. The fluorescence of 10,000 events was determined, and the data were analyzed using FlowJo VX software. Untreated cells served as a negative control.

### Fluorescence imaging

SKOV-3 and MCF-7 cells were grown at 37 °C in an atmosphere of 5% CO_2_ using Dulbecco's Modified Eagle and RPMI-1640 medium, respectively, supplemented with 10% fetal bovine serum and 0.1% penicillin-streptomycin. SKOV3 cells (4 × 10^4^ cells/well) and MCF7 (4 × 10^4^ cells/well) were seeded in the chamber and stabilized overnight in the corresponding media. In some experiments, the cells were incubated in media containing E-64 prior to treatment with Mil40-E-15C. Next, the cells were briefly washed with PBS and then treated with Mil40-E-15C or Mil40-E-15C, Lyso-Tracker and Mito-Tracker Red simultaneously in the media. After 8 or 24 h of incubation, the remaining Mil40-E-15C or organelle dyes were removed by washing three times with PBS, and the cells were placed in 1 mL of PBS solution. Fluorescence images were taken using a confocal laser scanning microscope (Zeiss LSM 510, Zeiss, Oberko, Germany).

### Animal experiments

All experiments were conducted in accordance with the Institutional Animal Care and Use Committee in a facility fully accredited by the Association for Assessment and Accreditation of Laboratory Animal Care (APU number: ON-CELL-XEN-06012020). For imaging studies, all animals were acclimatized to the animal facility for at least 48 h prior to experimentation. Each mouse weighed 20-25 g. In the *ex vivo* fluorescent imaging experiment, 0.2 mL of Mil40-E-15C (100 mg/kg) and PBS were administered to the mice via tail vein injection. Thirty hours after injection of Mil40-E-15C and PBS, the tumor tissues and various organs (lungs, heart, liver, kidneys and spleen) of the control and injected mice were dissected to obtain *ex vivo* images using a Maestro™ In-Vivo Fluorescence Imaging System. After *ex vivo* images were taken, the grafted tumor tissues were used to obtain cryosections in Tissue-Tek 100% Optimal Cutting Temperature Compound. The fresh tissue blocks were submerged in liquid nitrogen to ensure that the tissue was completely frozen and stored at -80 °C until sectioning. Subsequently, the blocks were cut with a microtome portion of the cryostat. Each section (thickness 10 μm) was placed on a glass slide for analysis using confocal fluorescent microscopy.

NOD-SCID female mice (6-8 weeks old) were used to establish the SKOV3 xenograft model, and CB17.SCID female mice (6-8 weeks) were used to establish the BT474 xenograft model. Upon tumor engraftment, mice were randomized to study groups (n = 6 each for mice treated with L-Histidine buffer, Mil40, Mil40 combined with MMAE, Mil40-E-15C and T-DM1 via tail vein injection) with each group averaging around 180 mm^3^ tumor volume in the SKOV3 xenograft model and 100 mm^3^ tumor volume in the BT474 xenograft model. Tumor size and body weight were monitored at least twice weekly, and tumor volume as a function of time was determined using the formula (L × W^2^)/2. The antitumor effect in the SKOV3 xenograft model was also assessed via histological examination of tumor section slides using H&E staining.

## Supplementary Material

Supplementary figures and tables.

## Abbreviations

ADCs: antibody-drug conjugates
FDA: food and drug administration
7-AHC: 7-amino-3-hydroxyethyl-coumarin
VA: valine-alanine
PAB: p-aminobenzyloxycarbonyl
CTSB: cathepsin B
VCit: valine citrulline
DIPEA: diisopropylethylamine
HIC: hydrophobic interaction chromatography
DAR: drug antibody ratio
PBS: phosphate-buffered saline
H&E: hematoxylin and eosin
