# Supplementary material for: A bifunctional molecule-based strategy for the development of theranostic antibody-drug conjugate
Source: Theranostics. 2021 Jan 1;11(6):2550–63. doi: 10.7150/thno.51232 (PMC7806464; doi:10.7150/thno.51232)
Supplement: Supplementary file 1 — Supplementary figures and tables. [file thnov11p2550s1.pdf]

Supporting Information

A bifunctional molecule-based strategy for development of theranostic antibody-drug conjugate

Dian Xiao,[†] Lei Zhao,[†] Fei Xie, Shiyong Fan, Lianqi Liu, Wei Li, Ruiyuan Cao, Song Li, Wu Zhong and Xinbo Zhou**

Table of Contents

1. Structure of coumarins and successful ADCs containing PAB

2. HIC, polymerization degree analysis of Mil40

3. Additional absorption and fluorescence data

4. Her2 expression in Her2 sensitive cell lines

5. Her2 expression in Her2 resistant cell line

6. Cytotoxicity of the theranostic ADC in cancer cell lines *in vitro*

7. Cell-cycle and apoptosis analyses of the theranostic ADC in tumor cell lines *in vitro*.

8. Cell cycle analysis of Mil40 and MMAE

9. Cell apoptosis analysis of Mil40 and MMAE

10. Supplementary figures of cell co-localization

11. Supplementary figures of In vivo antitumor efficacy in BT474 cell-inoculated xenograft mice.

12. ^{1}H-NMR, ^{13}C-NMR, ESI-MS Spectra

13. METHODS

1. Structures of coumarins and successful ADCs containing PAB

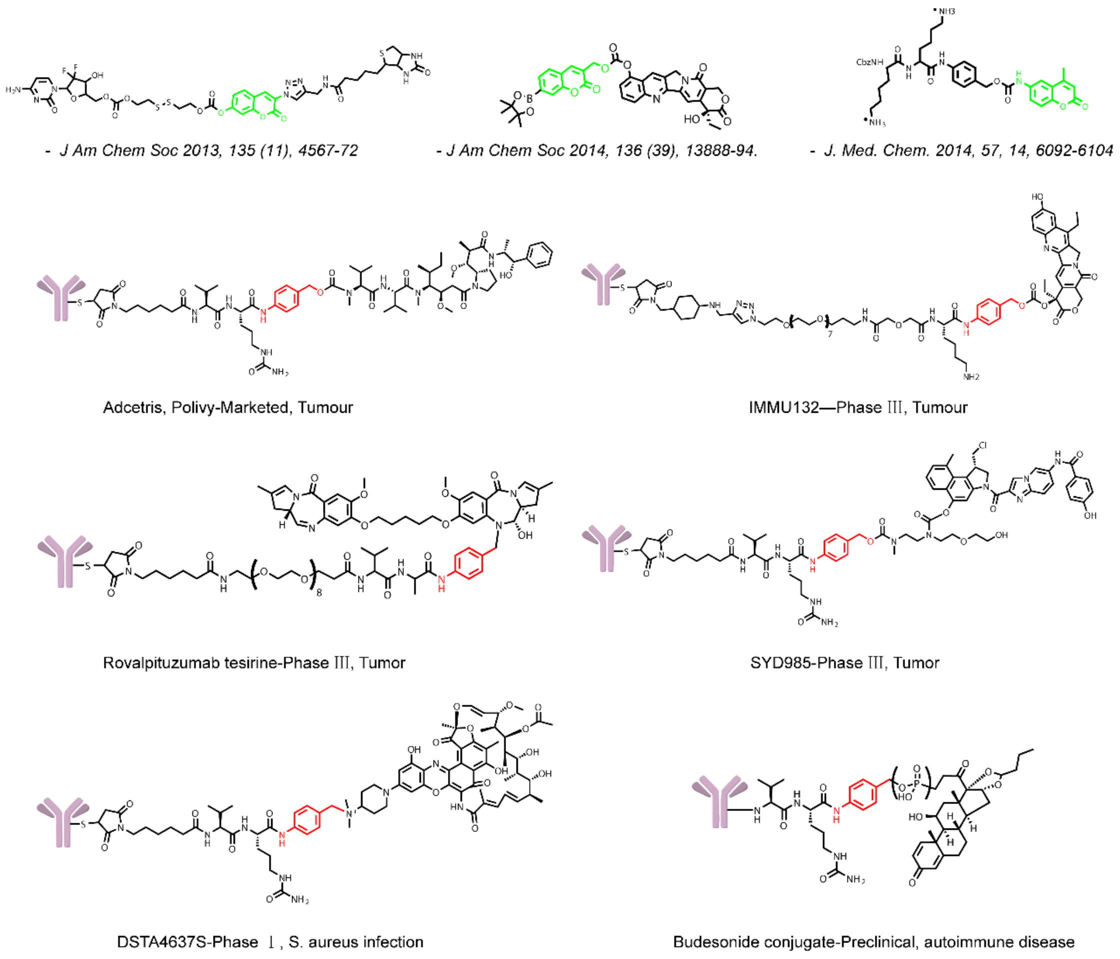

Figure S1. Structures of coumarins and successful ADCs containing PAB.

2. HIC, polymerization degree analysis of Mil40

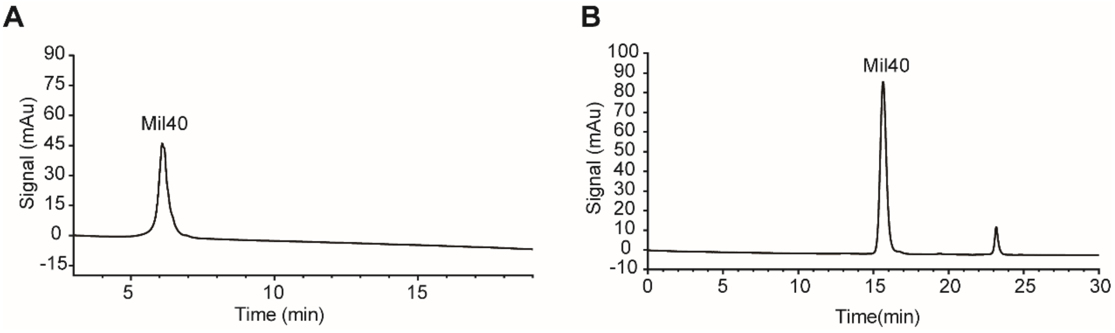

Figure S2. *In vitro* characterization of Mil40. (A) HIC analysis of Mil40. (B) Polymerization degree of Mil40.

3. Additional absorption and fluorescence data

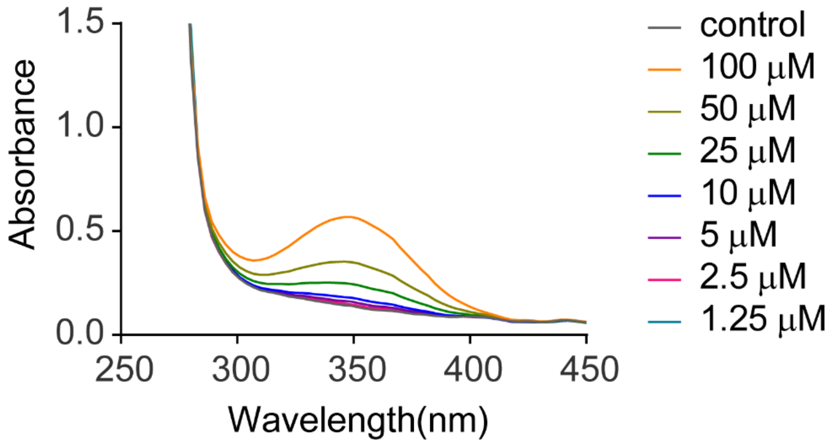

Figure S3. Absorption spectra of compound **3** at various concentrations.

4. HER2 expression in Her2 sensitive cell lines

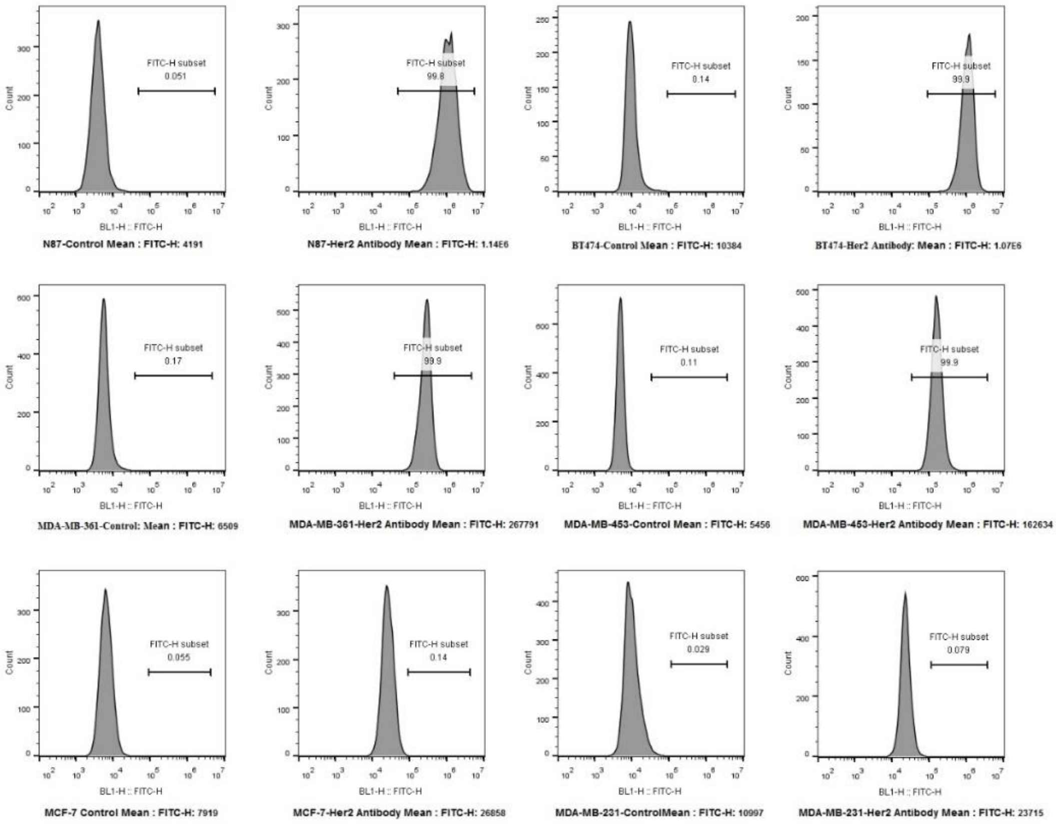

Figure S4. Her2 expression in Her2 sensitive cell lines, including N87, BT474, MDA-MB-361, MDA-MB-453, MCF-7 and MDA-MB-231.

5. HER2 expression in Her2 resistant cell line

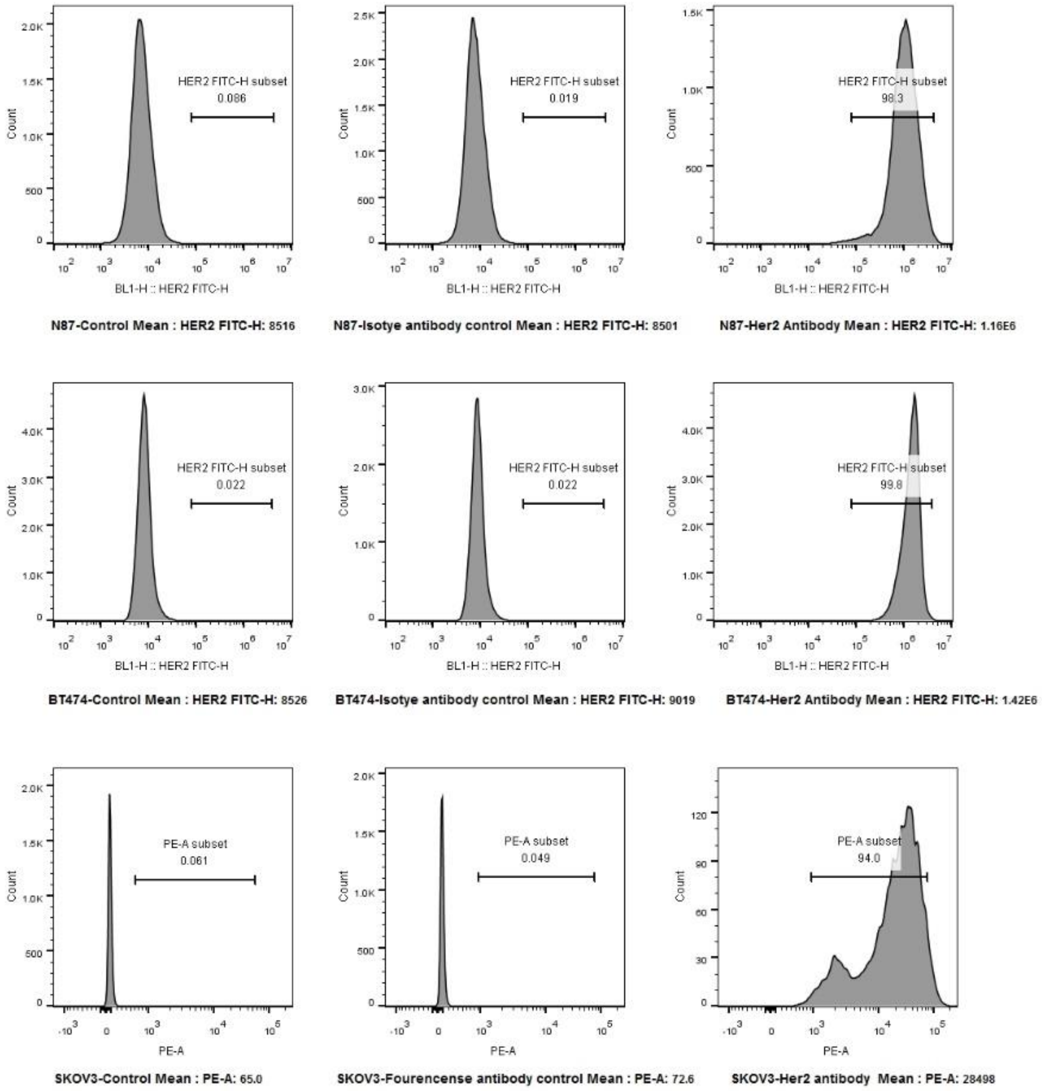

Figure S5. Her2 expression in Her2 resistant cell lines, including N87-HDR, BT474-HDR and SKOV3.

6. Cytotoxicity of the theranostic ADC in cancer cell lines *in vitro*

Table S1. Cytotoxicity of the theranostic ADC in cancer cell lines *in vitro*.

Cell lines	rMFI	Mil40-E-15C		mil40		MMAE		T-DM1	
		IC50 (nM)	Max. Inhibition	IC50 (nM)	Max. Inhibition	IC50 (nM)	Max. Inhibition	IC50 (nM)	Max. Inhibition
Herceptin sensitive cells									
NCI-N87	272.01	0.74	69.94%	>100	45.80%	0.55	68.22%	ND	ND
MDA-MB-361	41.14	0.11	86.23%	>100	33.41%	0.27	85.28%	ND	ND
MDA-MB-453	29.81	1.22	90.27%	314.5	34.83%	0.42	83.79%	ND	ND
MCF-7	3.39	32.48	53.87%	>100	-	1.93	80.33%	184.82	55.35%
MDA-MB-231	2.16	15.18	88.59%	240.04	40.84%	0.91	57.48%	ND	ND
Herceptin resistant cells									
BT474-HDR	157.45	0.58	94.84%	>100	20.75%	0.54	98.79%	0.46	78.32%
SKOV3	392.53	0.09	94.11%	>100	-	0.43	93.92%	0.24	95.78%
N87-HDR	136.45	3.74	64.31%	>100	-	0.75	70.19%	ND	ND

ND: Not detected. -: < 5% inhibition

7. Cell-cycle and apoptosis analyses of the theranostic ADC in tumor cell lines *in vitro*

Table S2. Cell-cycle and apoptosis analyses of the theranostic ADC in tumor cell lines *in vitro*.

Cell lines	rMFI	mil40-E-15C (10 nM)		mil40-E-15C (1 nM)		mil40-E-15C (0.1 nM)		mil40-E-15C (0.03nM)	
		G2/M%	Apoptosis%	G2/M%	Apoptosis%	G2/M%	Apoptosis%	G2/M%	Apoptosis%
BT474	103.04	61.68	81.4	37.06	48.1	12.95	33.7	10.60	17.0
NCI-N87	272.01	71.89	50.3	37.10	46.4	19.69	43.2	19.07	36.9

8. Cell cycle analysis of Mil40 and MMAE

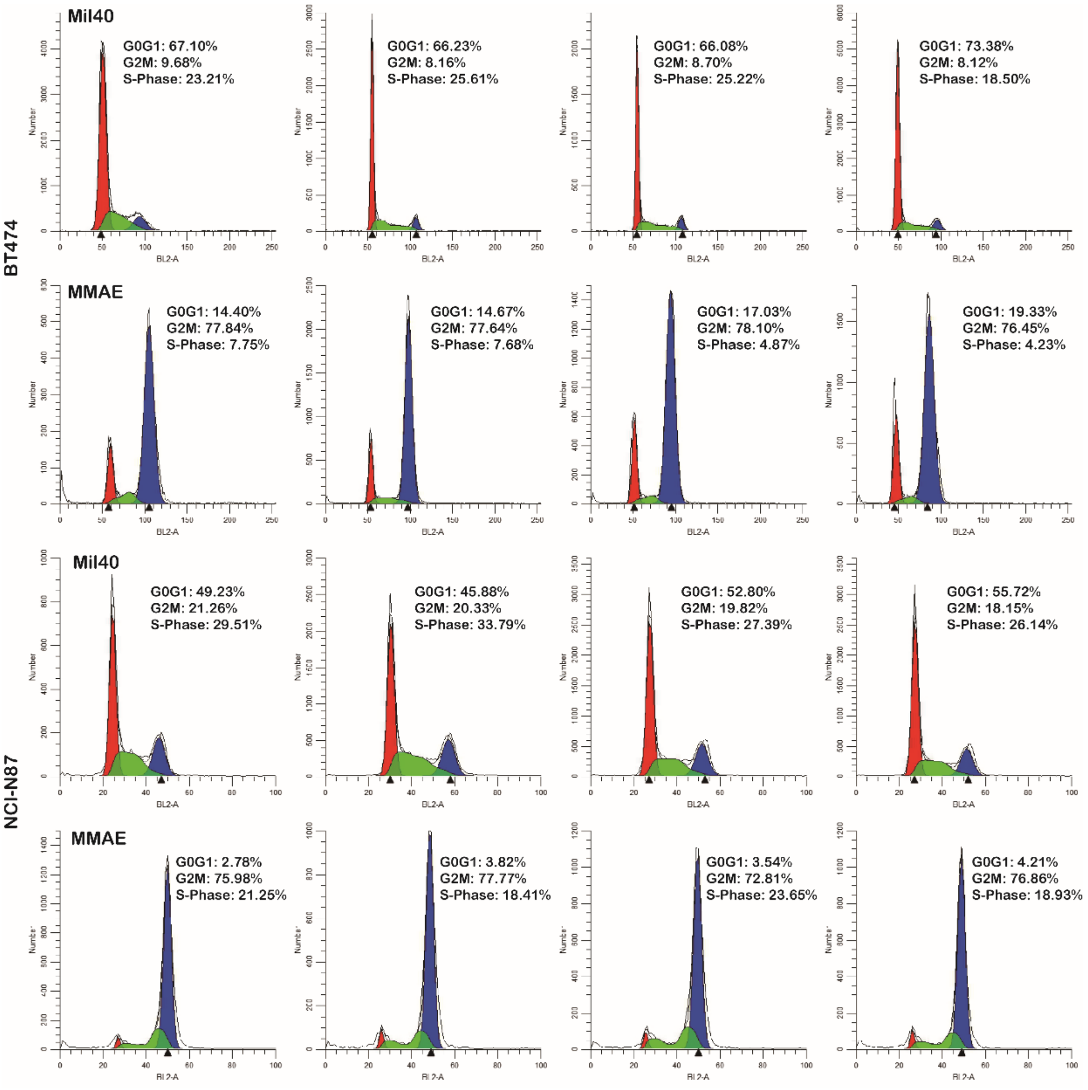

Figure S6. Cell-cycle analysis of Mil40 and MMAE under the concentration of 0.03 nM, 0.1 nM, 1 nM, 10 nM in BT474 and NCI-N87 cells.

9. Cell apoptosis analysis of Mil40 and MMAE

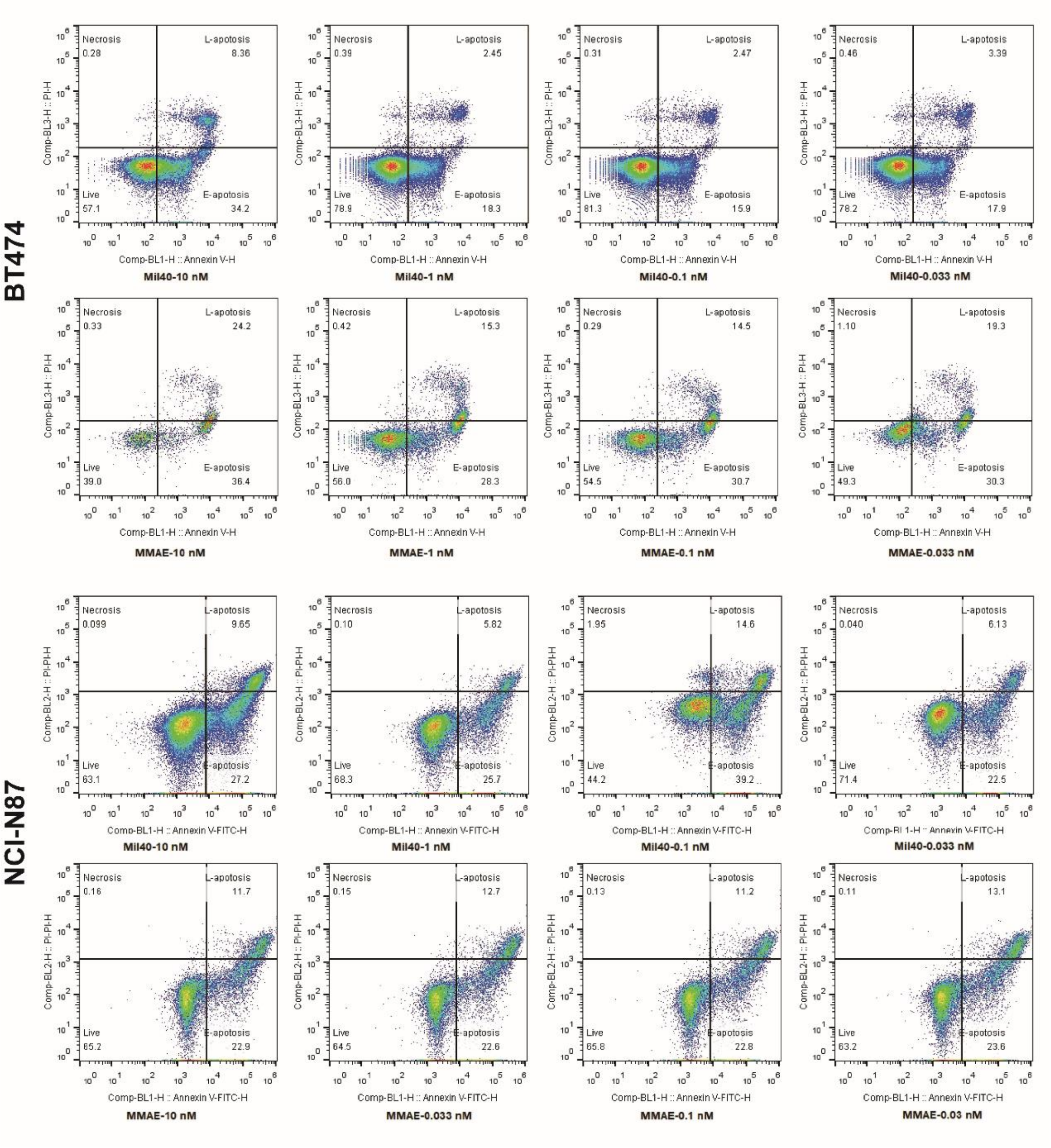

Figure S7. Cell apoptosis analysis of of Mil40 and MMAE under the concentration of 0.03 nM, 0.1 nM, 1 nM, 10 nM in BT474 and NCI-N87 cells.

10. Supplementary figures of cell co-localization

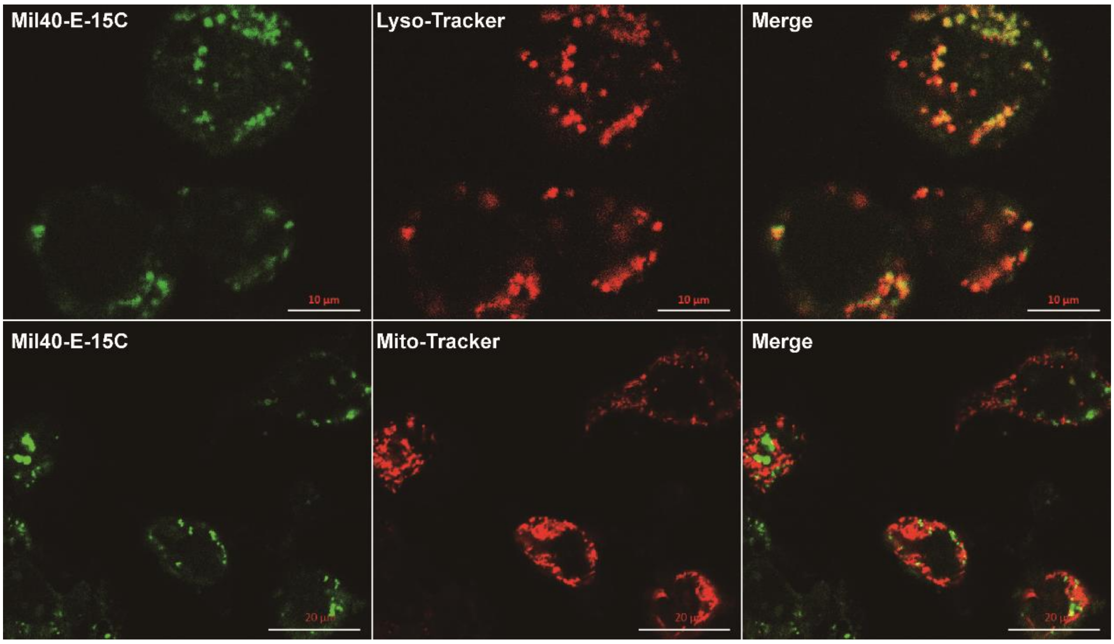

Figure S8. Supplementary figures of cell co-localization in SKOV3 cells.

11. Supplementary figures of *In vivo* antitumor efficacy in BT474 cell-inoculated xenograft mice

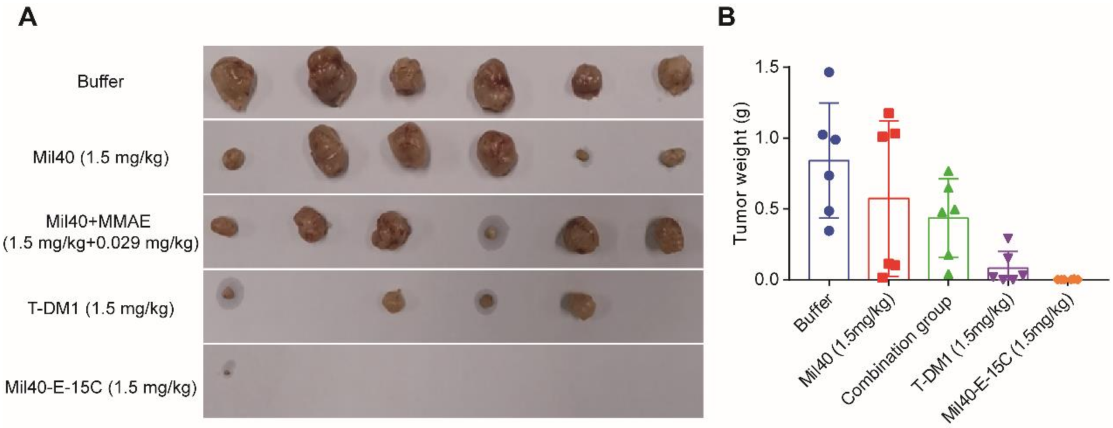

Figure S9. *In vivo* effects of Mil40-E-15C in BT474 cell-inoculated xenograft mice. (A) Resulting tumors excised from the animals after treatment on day 53. (B) Weight of resulting tumors on day 53.

12. ^{1}H-NMR, ^{13}C-NMR, ESI-MS Spectra

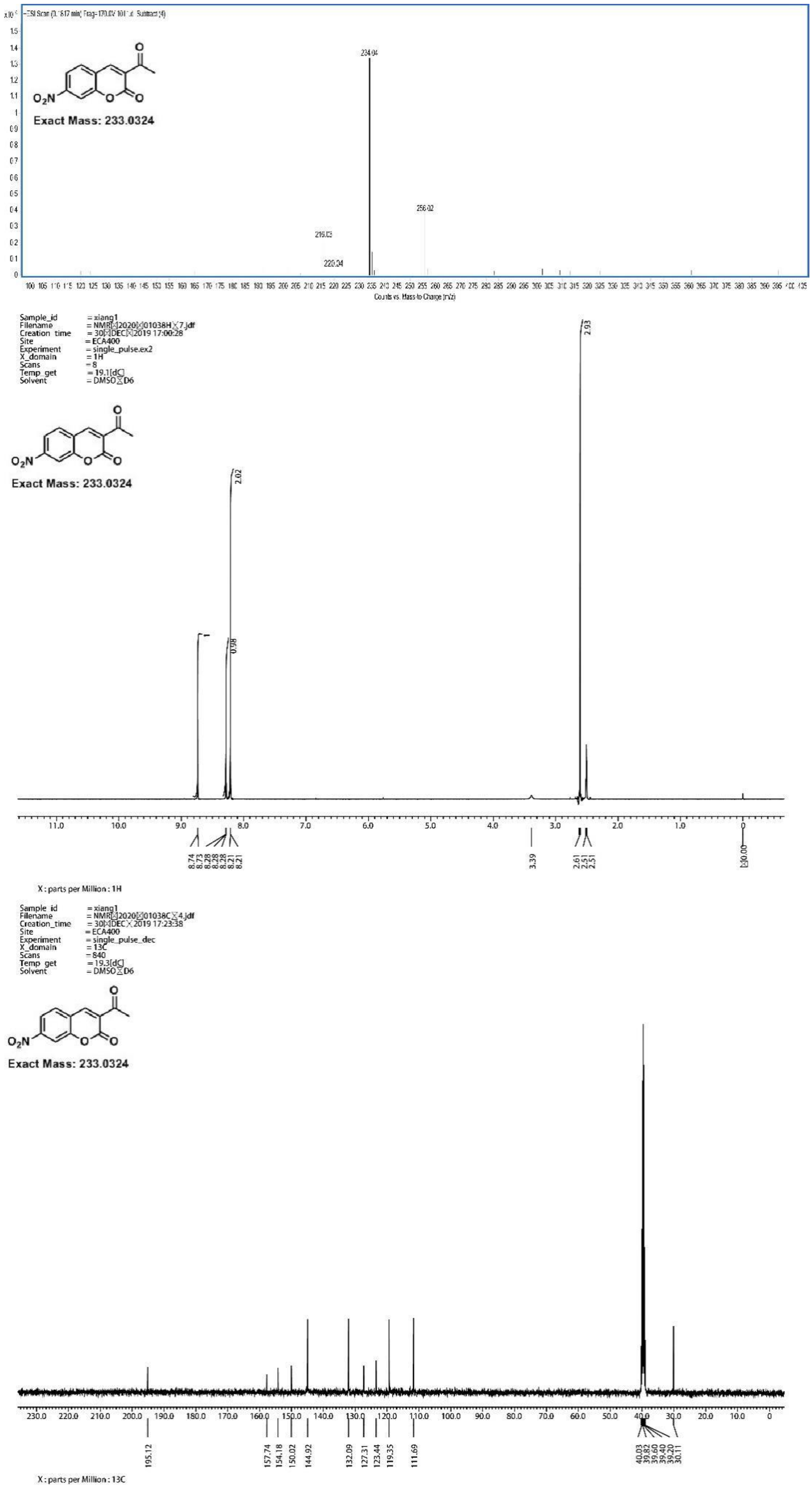

Figure S10. ^{1}H-NMR, ^{13}C-NMR, ESI-MS Spectra of intermediate **1.**

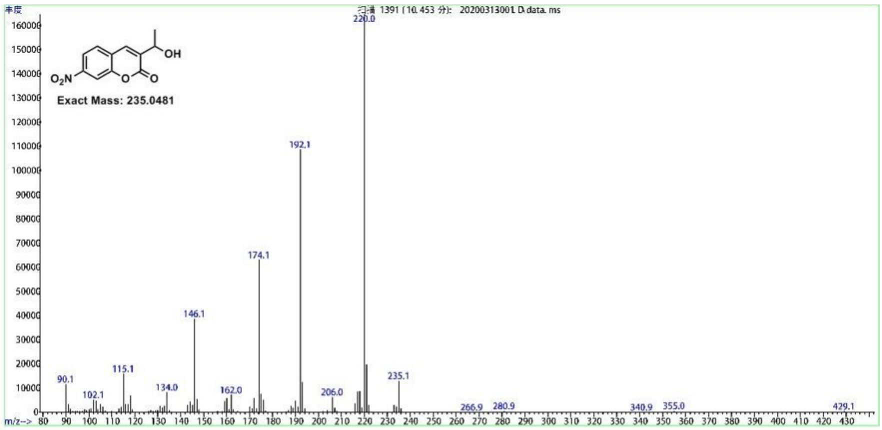

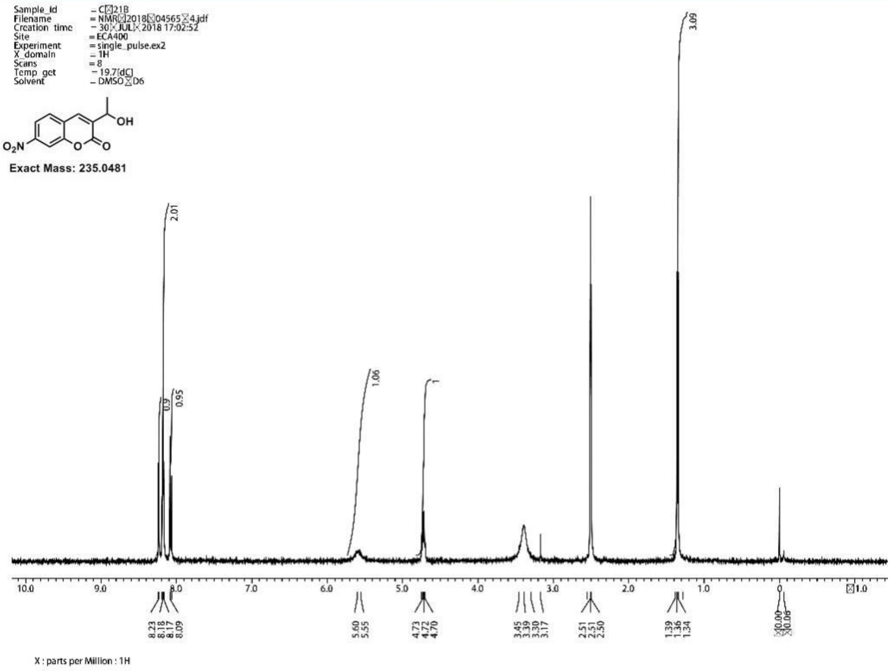

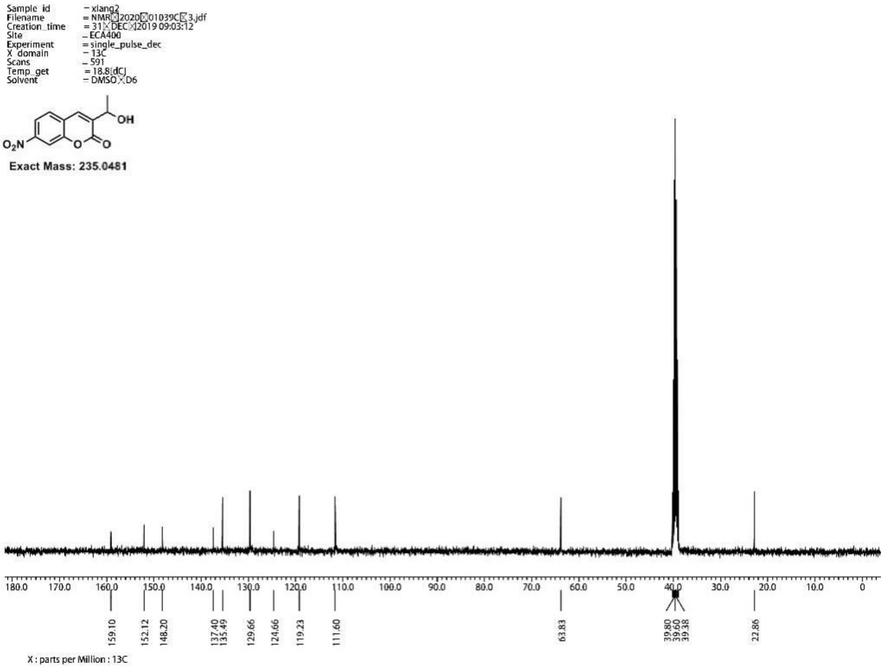

Figure S11. ^{1}H-NMR, ^{13}C-NMR, ESI-MS Spectra of intermediate **2.**

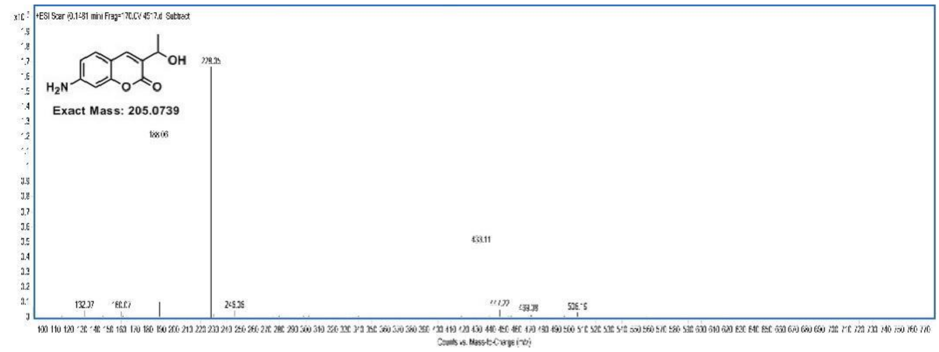

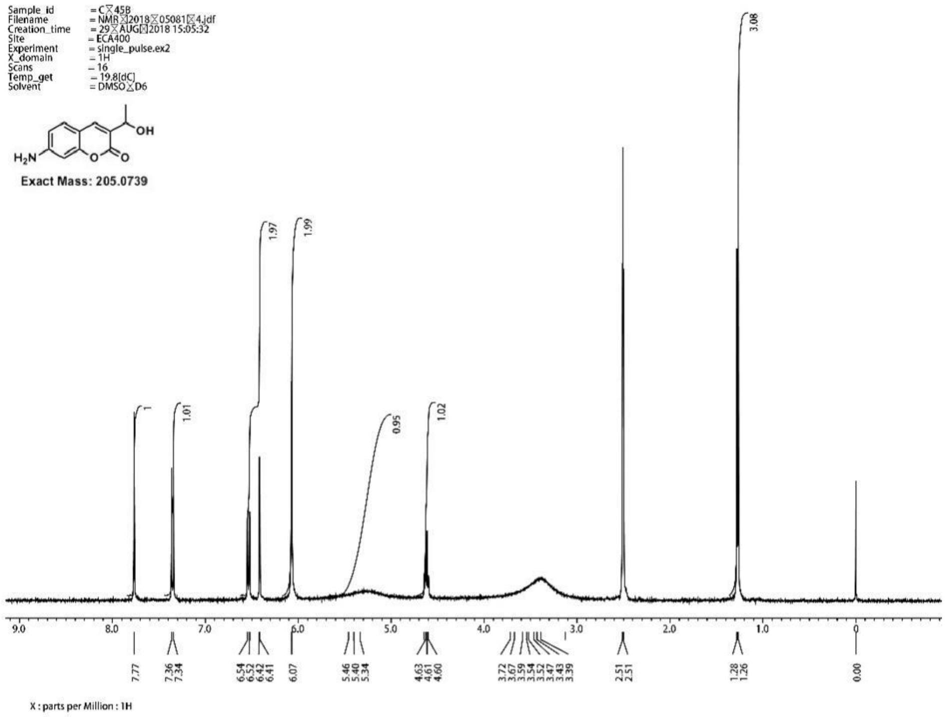

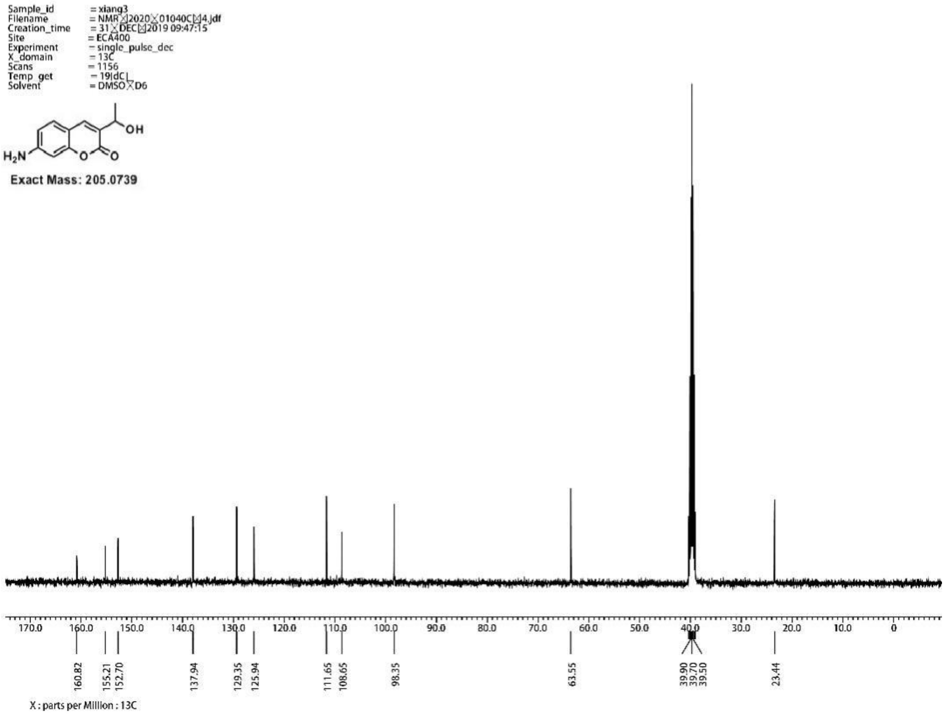

Figure S12. ^{1}H-NMR, ^{13}C-NMR, ESI-MS Spectra of intermediate **3.**

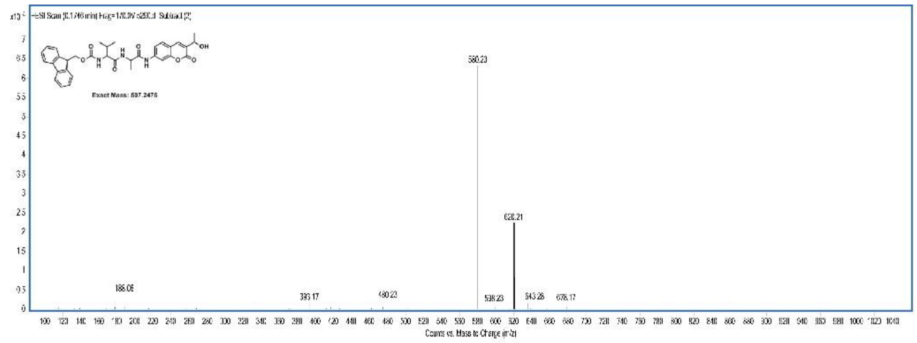

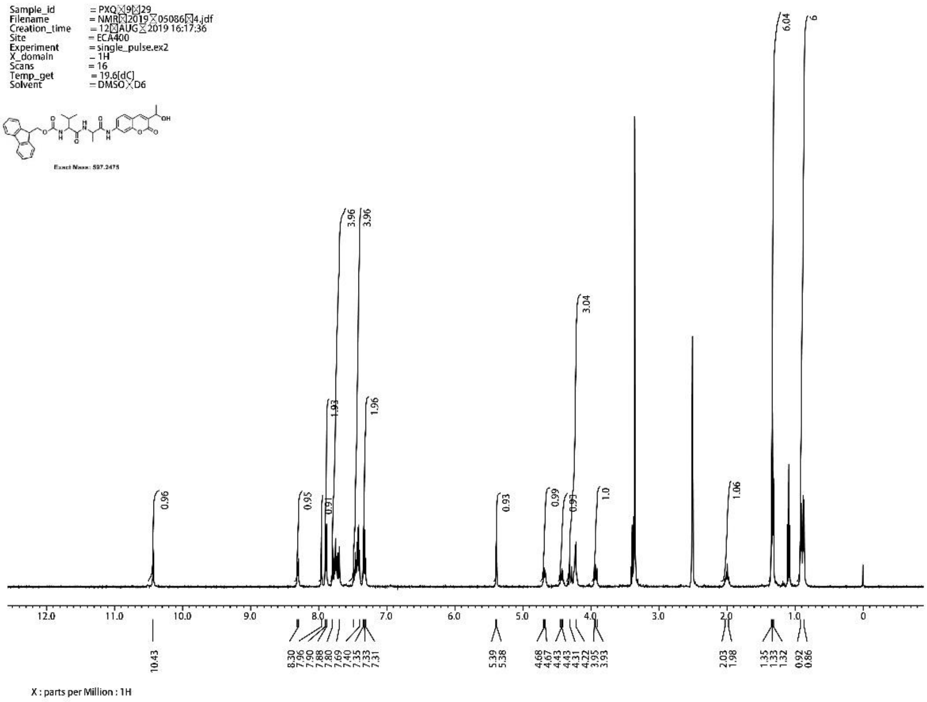

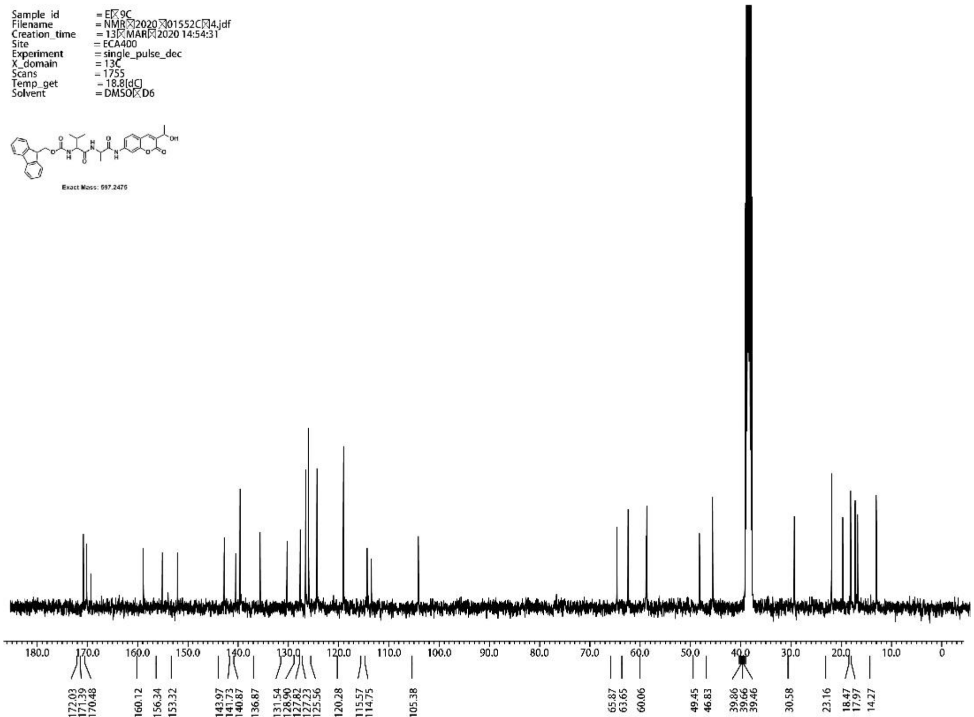

Figure S13. ^{1}H-NMR, ^{13}C-NMR, ESI-MS Spectra of intermediate **4.**

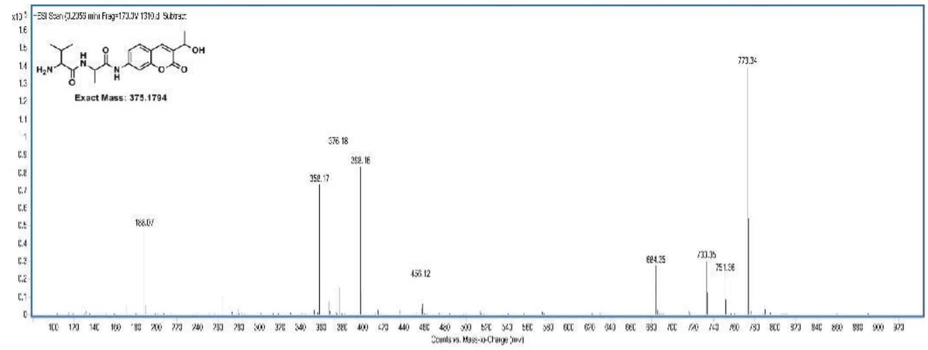

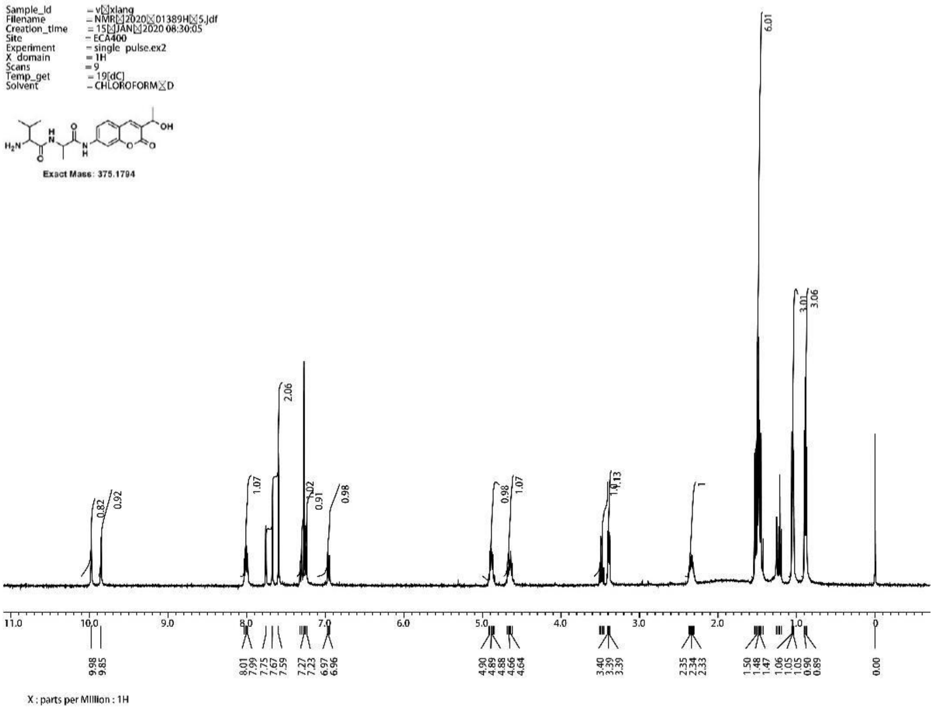

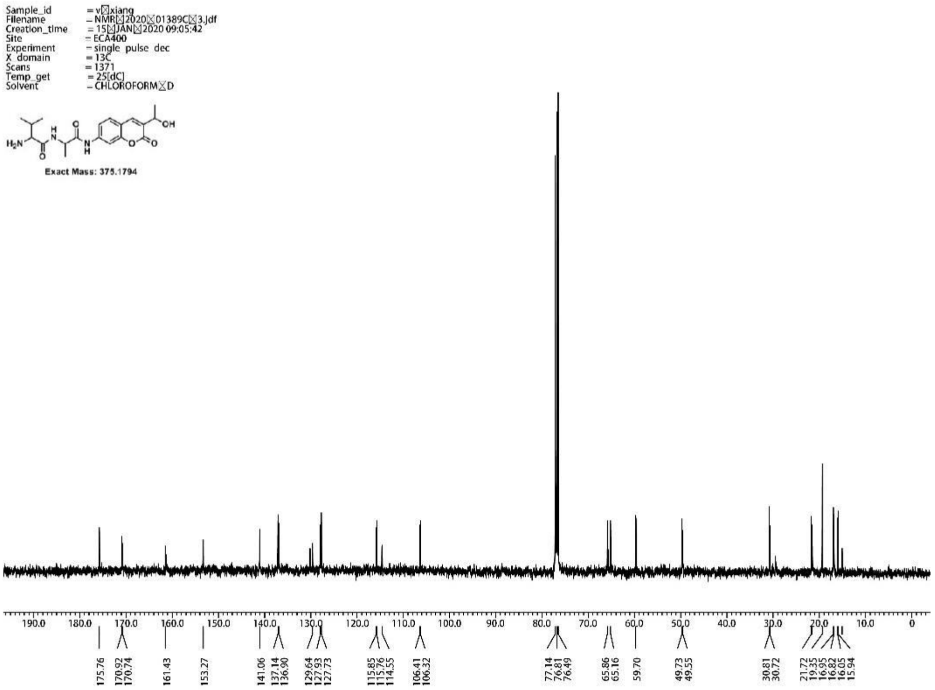

Figure S14. ^{1}H-NMR, ^{13}C-NMR, ESI-MS Spectra of intermediate **5.**

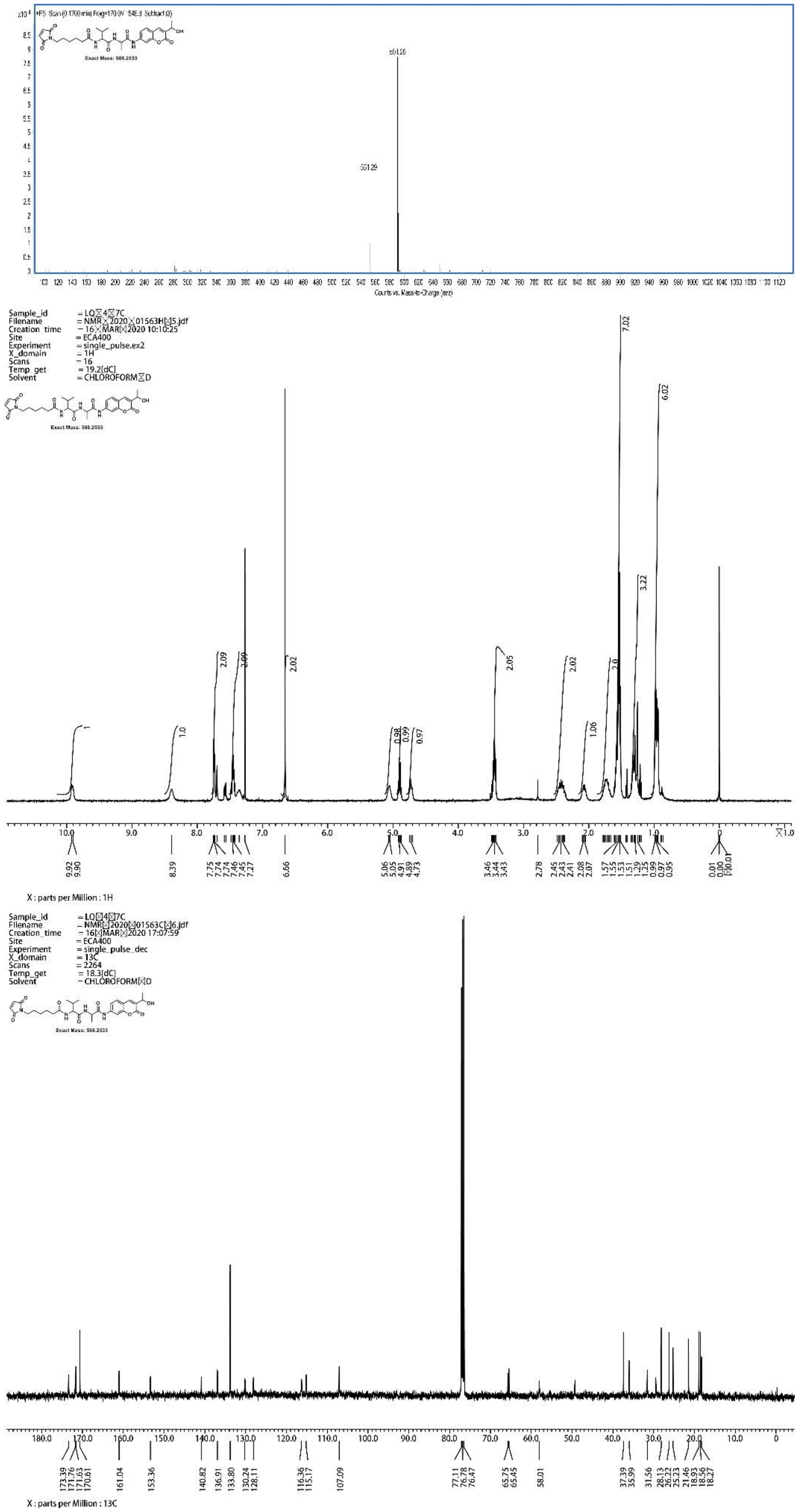

Figure S15. ^{1}H-NMR, ^{13}C-NMR, ESI-MS Spectra of intermediate **6.**

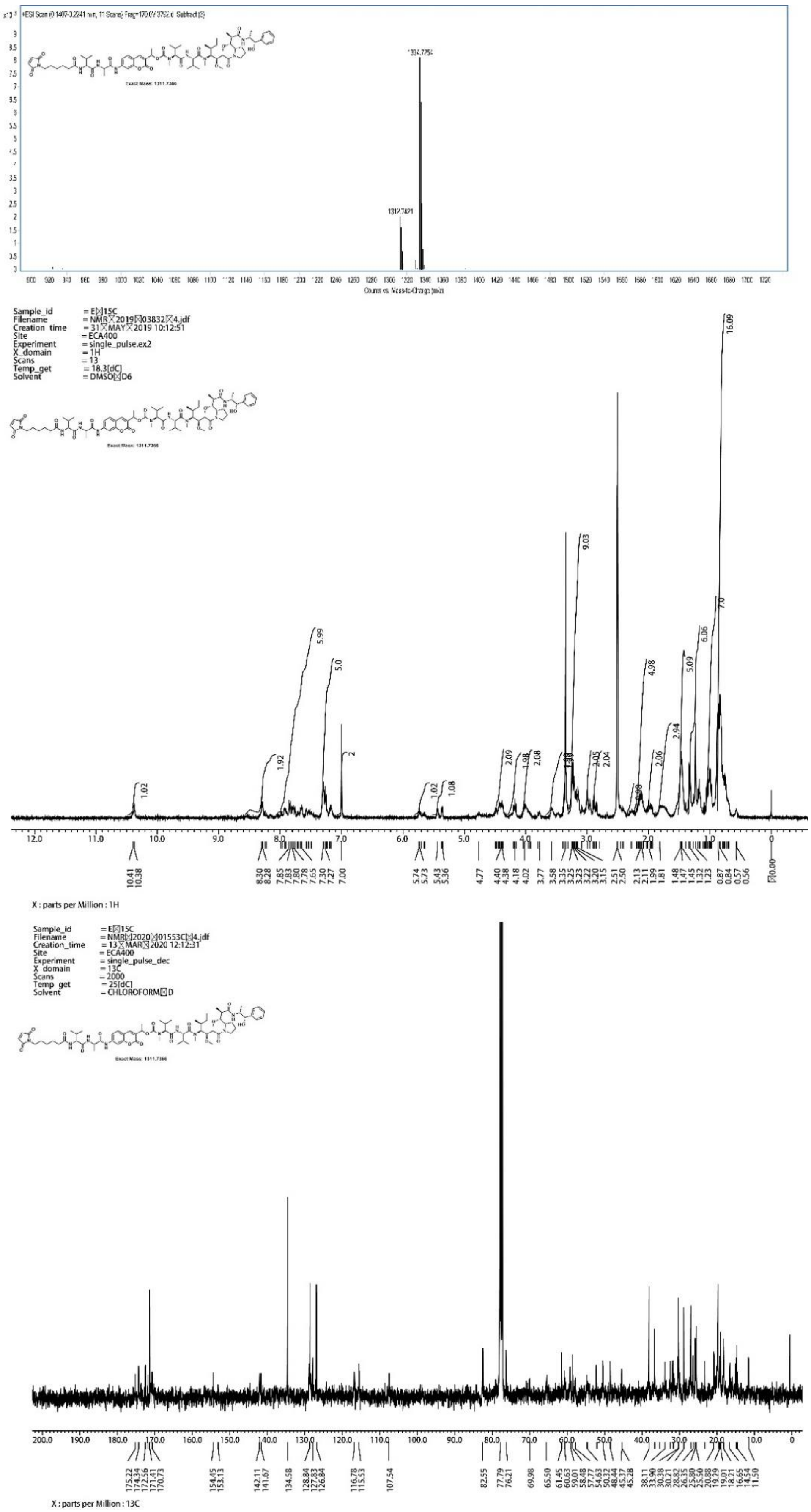

Figure S16. ^{1}H-NMR, ^{13}C-NMR, ESI-MS Spectra of compound **8.**

13. METHODS

Synthesis information.

Synthesis of 3-acetyl-7-nitro-2H-chromen-2-one (1). To a stirring mixture of 2-hydroxy-4-nitrobenzaldehyde (5.00 g, 30 mmol) and ethyl acetoacetate (4.6 mL, 36 mmol), 349 μL of piperidine were added. After reflux for 1.5 h, the yellowish solid separated was filtered off, subsequently washed with ethanol to afford intermediate 1 (4.00 g, 57.1% yield). ^{1}H NMR (DMSO−D6, 400 MHz): δ 8.74 – 8.73 (d, *J* = 4 Hz, 1H), 8.29 – 8.28 (m, 1H), 8.22 – 8.21 (d, *J* = 4 Hz, 2H), 2.61 (s, 3H). ^{13}C NMR (DMSO−D6, 400 MHz): δ 195.12, 157.74, 154.18, 150.02, 144.92, 132.09, 127.31, 123.44, 119.35, 111.69, 30.11. MS m/z $[M + H]^+$ calculated for $C_{11}H_7NO_5$: 234.03, found: 234.04.

Synthesis of 3-(1-hydroxyethyl)-7-nitro-2H-chromen-2-one (2). To a solution of intermediate **1** (2.40 g, 10.30 mmol) in methanol and tetrahydrofuran (1:1, 200 mL total) was added sodium borohydride (390 mg, 10.30 mmol) and cerium chloride (2.54 g, 10.3 mmol) at 0°C. After completion of the reaction within 1.5 h, the solvent was concentrated in vacuo and the crude product was purified by column chromatography (1:1.5 EtOAc/hexanes) to give the desired intermediate **2** as yellow solid (1.80 g, 74.3 % yield). ^{1}H NMR (DMSO−D6, 400 MHz): δ 8.24 – 8.23 (d, *J* = 4 Hz, 1H), 8.19 – 8.17 (m, 2H), 8.09 – 8.06 (d, *J* = 12 Hz, 1H), 5.60 – 5.55 (br, 1H), 4.75 – 4.70 (q, *J* = 4 Hz, 1H), 1.36 – 1.34 (d, *J* = 8 Hz, 3H). ^{13}C NMR (DMSO−D6, 400 MHz): δ 159.11, 152.13, 148.20, 137.40, 135.50, 129.67, 124.66, 119.24, 111.61, 63.84, 22.86. MS m/z $[M]^+$ calculated for $C_{11}H_9NO_5$: 235.05, found: .235.1.

Synthesis of 7-amino-3-(1-hydroxyethyl)-2H-chromen-2-one (3). Intermediate **2** (500 mg, 2.13 mmol), Iron(III) chloride hexahydrate (115 mg, 0.45 mmol), hydrazine hydrate (1.50 g, 25.6 mmol) and active carbon (305 mg, 25.6 mmol) were mixed in absolute ethanol (30 mL) and refluxed for 2 h. The solution was filtered and the filtrate was concentrated in vacuo and the crude product was purified by column chromatography (1:1 EtOAc/hexanes) to yield **3** as the white solid (300 mg, 68.8 % yield). ^{1}H NMR (DMSO−D6, 400 MHz): δ 7.77 (s, 1H), 7.36 – 7.34 (d, *J* = 8 Hz, 1H), 6.55 – 6.52 (dd, *J* = 4 Hz, 1H), 6.42 – 6.41 (d, *J* = 4 Hz, 1H), 6.07 (s, 2H), 5.46 – 5.34 (br, 1H), 4.65 – 4.60 (q, *J* = 4 Hz, 1H), 1.28 – 1.26 (d, *J* = 8 Hz, 1H). ^{13}C NMR (DMSO−D6, 400 MHz): δ 160.82, 155.21, 152.70, 137.94, 129.35, 125.94, 111.65, 108.65, 98.35, 63.55, 23.44. MS m/z $[M + Na]^+$ calculated for $C_{11}H_{11}NO_3$: 228.07, found: 228.06.

Synthesis of (9H-fluoren-9-yl)methyl (1-((1-((3-(1-hydroxyethyl)-2-oxo-2H-chromen-7-yl)amino)-1-oxopropan-2-yl)amino)-3-methyl-1-oxobutan-2-yl)carbamate (4). Fmoc-valine-alanine (400 mg, 0.98 mmol) and 4-Methylmorpholine (132 µL, 1.18mmol) was dissolved in 18 mL dry tetrahydrofuran, T_3P (750 mg, 2.34 mmol) in ethyl acetate was slowly added. The reaction mixture was kept under 0°C and stirred for 30 min. Then intermediate **3** in 5 mL dry tetrahydrofuran was slowly added to the solution. The reaction was stirred at room temperature for 3 h. After completion of the reaction, the solvent was concentrated in vacuo and the crude product was purified by column chromatography (1.5:1 EtOAc/hexanes) to give the desired intermediate **4** as white solid (430 mg, 73.8 % yield). ^{1}H NMR (DMSO−D6, 400 MHz):

10.43 (s, 1H), 8.31 – 8.30 (d, *J* = 4 Hz, 1H), 7.96 (s, 1H), 7.90 – 7.88 (d, *J* = 8 Hz, 2H), 7.80 – 7.69 (m, 4H), 7.49 – 7.40 (m, 4H), 7.35 – 7.31 (t, *J* = 8 Hz, 2H), 5.39 – 5.38 (d, *J* = 4 Hz, 1H), 4.69 – 4.67 (t, *J* = 4 Hz, 1H), 4.44 – 4.42 (t, *J* = 4 Hz, 1H), 4.31 – 4.22 (m, 3H), 3.95 – 3.91 (t, *J* = 8 Hz, 1H), 2.03 – 1.98 (m, 1H), 1.35– 1.31 (t, *J* = 8 Hz, 6H), 0.92 – 0.86 (m, 6H). ^{13}C NMR (DMSO−D6, 400 MHz): δ 172.04, 171.40, 170.48, 160.12, 156.35, 153.32, 143.97, 141.73, 140.87, 140.87, 136.87, 131.54, 128.90, 127.82, 127.82, 127.23, 127.23, 125.56, 125.56, 120.28, 120.28, 115.57, 114.75, 105.38, 65.87, 63.65, 60.06, 49.45, 46.83, 30.58, 23.16, 18.47, 17.97, 14.27. MS m/z $[M + H]^+$ calculated for $C_{34}H_{35}N_3O_7$: 597.24, found: 598.23.

Synthesis of 2-amino-N-(1-((3-(1-hydroxyethyl)-2-oxo-2H-chromen-7-yl)amino)-1-oxopropan-2-yl)-3-methylbutanamide (5). Intermediate **4** (410 mg, 0.69 mmol) was dissolved in 10 mL dry N,N-Dimethylformamide, piperidine (500 μL) was slowly added. The reaction was stirred at room temperature for 30 min. After completion of the reaction, the solvent was concentrated in vacuo and the crude product was purified by column chromatography (15:1 DCM/CH_3OH) to give the desired intermediate **5** as yellow solid (220 mg, 85.6 % yield). ^{1}H NMR (DMSO−D6, 400 MHz): 9.98 (s, 1H), 9.85 (s, 1H), 8.01 (t, *J* = 8 Hz, 1H), 7.76 – 7.59 (m, 2H), 7.32 – 7.30 (m, 2H), 7.26 – 7.23 (m, 2H), 6.97– 6.94 (dd, *J* = 4 Hz, 1H), 4.92 – 4.85 (m, 1H), 4.68 – 4.62 (m, 1H), 3.51 – 3.46 (q, *J* = 8 Hz, 1H), 3.40 – 3.38 (m, 1H), 2.36 – 2.31 (m, 1H), 1.54 – 1.43 (m, 6H), 1.06 – 1.04 (m, 3H), 0.90 – 0.87 (m, 3H). ^{13}C NMR (DMSO−D6, 400 MHz): δ 175.76, 170.92, 161.43, 153.27, 141.06, 137.14, 129.64, 127.93, 115.85, 114.55,

106.41, 65.86, 59.70, 49.73, 30.81, 21.72, 19.35, 16.82, 16.05. MS m/z $[M + H]^+$ calculated for $C_{19}H_{25}N_3O_5$: 376.18, found: 376.18.

Synthesis of 6-(2,5-dioxo-2,5-dihydro-1H-pyrrol-1-yl)-N-(1-((1-((3-(1-hydroxyethyl)-2-oxo-2H-chromen-7-yl)amino)-1-oxopropan-2-yl)amino)-3-methyl-1-oxobutan-2-yl)hexanamide (6). Intermediate **5** (186 mg, 0.50 mmol) was dissolved in 7 mL dry N,N-Dimethylformamide, 6-maleimidohexanoic acid N-hydroxysuccinimide ester (305 mg, 0.99 mmol) and DIPEA (77 mg, 0.60 mmol) was slowly added. The reaction was stirred at room temperature overnight. After completion of the reaction, the solvent was concentrated in vacuo and the crude product was purified by column chromatography (15:1 DCM/CH_3OH) to give the desired intermediate **6** as white solid (270 mg, 96.4 % yield). ^{1}H NMR ($CDCl_3$–D6, 400 MHz): 9.92 (br, 1H), 8.39 (br, 1H), 7.75 – 7.70 (m, 2H), 7.48 – 7.42 (m, 2H), 6.66 (s, 2H), 5.06 (br, 1H), 4.92 – 4.87 (q, J = 8 Hz, 1H), 4.75 – 4.71 (t, J = 8 Hz, 1H), 3.46 – 3.43 (t, J = 8 Hz, 2H), 2.48 – 2.37 (m, 2H), 2.10 – 2.05 (m, 1H), 1.78 – 1.67 (m, 2H), 1.61 – 1.51 (m, 7H), 1.35 – 1.21 (m, 3H), 0.99 – 0.94 (m, 6H). ^{13}C NMR ($CDCl_3$–D6, 400 MHz): δ 173.39, 171.76, 171.63, 170.61, 170.61, 161.04, 153.36, 140.82, 136.91, 133.80, 133.80, 130.24, 128.11, 116.36, 115.17, 107.09, 65.75, 65.45, 58.01, 37.39, 35.99, 31.56, 28.13, 26.22, 25.23, 21.46, 18.93, 18.56, 18.27. MS m/z $[M + Na]^+$ calculated for $C_{29}H_{36}N_4O_8$: 591.24, found: 591.29.

Synthesis of 1-(7-(2-(2-(6-(2,5-dioxo-2,5-dihydro-1H-pyrrol-1-yl)hexanamido)-3-methylbutanamido)propanamido)-2-oxo-2H-chromen-3-yl)ethyl (4-nitrophenyl) carbonate (7). Under argon protection, to a 5 mL aqueous of intermediate **6** (230 mg,

0.41 mmol) and bis(4-nitrophenyl) carbonate (246 mg, 0.81 mmol) was added DIPEA (63 mg, 0.49 mmol). The reaction was stirred at room temperature overnight. After completion of the reaction, to the residual brown syrup was added ethyl acetate (10 ml) and stirred for 30 min, the precipitate was filtered to yield **7** as a yellow solid (210 mg, 70.9 % yield). The precipitate was not purified and proceeded to the next step directly.

Synthesis of 1-(7-(2-(2-(6-(2,5-dioxo-2,5-dihydro-1H-pyrrol-1-yl)hexanamido)-3-methylbutanamido)propanamido)-2-oxo-2H-chromen-3-yl)ethyl ((S)-1-(((S)-1-(((3R,4S,5S)-1-((S)-2-((1R,2R)-3-(((1S,2R)-1-hydroxy-1-phenylpropan-2-yl)amino)-1-methoxy-2-methyl-3-oxopropyl)pyrrolidin-1-yl)-3-methoxy-5-methyl-1-oxoheptan-4-yl)(methyl)amino)-3-methyl-1-oxobutan-2-yl)amino)-3-methyl-1-oxobutan-2-yl)(methyl)carbamate (8). Under argon protection, to a 5 mL solution of intermediate **7** (230 mg, 0.41 mmol), MMAE (187 mg, 0.26 mmol) and HOBT (42 mg, 0.31 mmol) was added DIPEA (67 mg, 0.52 mmol). The reaction was stirred at room temperature overnight. After completion of the reaction, the solvent was concentrated in vacuo and the crude product was purified by column chromatography (15:1 DCM/CH_3OH) to give the desired compound **8** as white solid (100 mg, 29.9 % yield). ^{1}H NMR (DMSO−D6, 400 MHz): 10.41 – 10.28 (d, J = 12 Hz, 1H), 8.30 – 8.22 (m, 2H), 7.85 – 7.65 (m, 6H), 7.30 – 7.25 (m, 5H), 7.00 (s, 2H), 5.74 – 5.64 (m, 1H), 5.43 – 5.36 (m, 1H), 4.50 – 4.37 (m, 2H), 4.20 – 4.16 (t, J = 8 Hz, 2H), 4.04 – 3.92 (m, 2H), 3.58 (br, 2H), 3.39 – 3.37 (m, 2H), 3.25 – 3.15 (m, 9H), 3.00 – 2.95 (m, 2H), 2.90 – 2.80 (m, 2H), 2.30 – 2.27 (m, 1H), 2.18 – 2.08 (m, 5H), 2.03 – 1.95 (m, 2H), 1.81 (br, 3H), 1.48 – 1.45 (m, 5H), 1.34 – 1.16 (m, 6H), 1.11 – 0.97 (m, 7H), 0.87 – 0.73 (m,

16H). ^{13}C NMR ($CDCl_3$–D6, 400 MHz): δ 175.22, 174.34, 174.34, 172.56, 172.56, 171.81, 171.41, 171.41, 171.41, 170.73, 154.45, 153.13, 142.11, 141.67, 134.58, 134.58, 134.58, 128.84, 128.58, 128.58, 128.58, 127.83, 126.84, 126.84, 116.78, 115.33, 107.54, 82.55, 77.79, 76.21, 69.98, 65.20, 61.45, 60.63, 59.01, 58.48, 57.77, 54.63, 52.03, 50.32, 48.44, 45.37, 45.28, 38.11, 36.61, 33.90, 32.45, 31.63, 30.38, 30.21, 28.82, 26.91, 26.35, 25.80, 25.50, 23.23, 20.88, 19.56, 19.56, 19.56, 19.56, 19.29, 19.01, 18.21, 18.21, 16.65, 14.89, 14.65, 11.50. HR – MS (ESI+) m/z $(M+H)^+$ calculated for $C_{69}H_{101}N_9O_{16}$, 1312.7366; found: 1312.7421.
